# Null diffusion-based enrichment for metabolomics data

**DOI:** 10.1371/journal.pone.0189012

**Published:** 2017-12-06

**Authors:** Sergio Picart-Armada, Francesc Fernández-Albert, Maria Vinaixa, Miguel A. Rodríguez, Suvi Aivio, Travis H. Stracker, Oscar Yanes, Alexandre Perera-Lluna

**Affiliations:** 1 Departament d’Enginyeria de Sistemes, Automàtica i Informàtica Industrial, Universitat Politècnica de Catalunya, Barcelona, Spain; 2 Networking Biomedical Research Centre in the subject area of Bioengineering, Biomaterials and Nanomedicine (CIBER-BBN), Madrid, Spain; 3 Institut de Recerca Pediàtrica Hospital Sant Joan de Déu, Esplugues de Llobregat, Barcelona, Spain; 4 Centre for Omic Sciences, Rovira i Virgili University, Reus, Spain; 5 Department of Electronic Engineering, Rovira i Virgili University, Tarragona, Spain; 6 Metabolomics Platform, Spanish Biomedical Research Centre in Diabetes and Associated Metabolic Disorders, Madrid, Spain; 7 Institute for Research in Biomedicine, Barcelona Institute of Science and Technology, Barcelona, Spain; 8 Takeda Cambridge Ltd, Cambridge, United Kingdom; University of Alberta, CANADA

## Abstract

Metabolomics experiments identify metabolites whose abundance varies as the conditions under study change. Pathway enrichment tools help in the identification of key metabolic processes and in building a plausible biological explanation for these variations. Although several methods are available for pathway enrichment using experimental evidence, metabolomics does not yet have a comprehensive overview in a network layout at multiple molecular levels. We propose a novel pathway enrichment procedure for analysing summary metabolomics data based on sub-network analysis in a graph representation of a reference database. Relevant entries are extracted from the database according to statistical measures over a null diffusive process that accounts for network topology and pathway crosstalk. Entries are reported as a sub-pathway network, including not only pathways, but also modules, enzymes, reactions and possibly other compound candidates for further analyses. This provides a richer biological context, suitable for generating new study hypotheses and potential enzymatic targets. Using this method, we report results from cells depleted for an uncharacterised mitochondrial gene using GC and LC-MS data and employing KEGG as a knowledge base. Partial validation is provided with NMR-based tracking of ^13^C glucose labelling of these cells.

## Introduction

Metabolomics is the science that studies the chemical reactions taking place in a living organism by measuring their lightweight reactants and products, also called metabolites. Metabolomics is used in the study of human disease, biomarker identification, drug evaluation and treatment prognosis [1]. Metabolomics datasets are generated from the identification and quantification of the metabolites in a sample. Afterwards, statistical analysis of the datasets enables researchers to devise a plausible explanation for the changes identified and to understand the underlying biological processes involved [2].

Current methods to measure metabolites mainly rely on Nuclear Magnetic Resonance (NMR) and Mass Spectrometry (MS) technologies [3], the latter consisting of two broad categories: Liquid Chromatography and Gas Chromatography coupled to MS (LC/MS and GC/MS). Raw data processing, also known as primary analysis, can be achieved using tools including MeltDB [4], MetaboAnalyst [5], MAIT [6], along with spectral databases [7] like the Human Metabolome Database [8], resulting in a table of relative metabolite abundances.

Data interpretation, known as secondary analysis, benefits from the identification of metabolic pathways to draw conclusions, encouraging the use of so-called pathway enrichment techniques. Their purpose is to provide the metabolites with their biological context, drawing from comprehensive databases like Kyoto Encyclopedia of Genes and Genomes, KEGG [9], Reactome [10], WikiPathways [11] and the Small Molecule Pathway Database [11]. Enrichment outputs can be further analysed by manual network manipulation through tools such as Cytoscape [12], whose plug-in MetScape [13] builds networks containing compounds, reactions, enzymes and genes. In this work, pathway enrichment techniques will be divided into three generations, following the review in [14].

The first generation of enrichment techniques is based on Over Representation Analysis (ORA), a statistical test that assesses whether the occurrence of a label within a subset is greater than expected by chance in the background population. Applied to metabolomics, it takes as input the identifiers of affected metabolites (previously determined through a statistical test involving conditions) and assesses a p-value for each pathway. ORA is available through the web tools IMPaLA [15], MetaboAnalyst, MBRole and MPEA [16, 17]. Limitations of ORA include an oversimplification of the biology, a thresholding decision issue when generating the input metabolite list and a lower power for capturing subtle and coordinated changes within a pathway [18].

A second generation of enrichment methods, Functional Class Scoring (FCS), avoids the cutoff choice in generating the affected metabolite list and claims the capability of capturing subtle but consistent changes in concentration [2, 19]. This concept was imported from Gene Set Enrichment Analysis [18] and is available through MSEA [20] in MetaboAnalyst and IMPaLA. A shortcoming of FCS methods is that they ignore the network nature of biological pathways [14]. As biological datasets are heterogeneous, and as no method is always best, the researcher’s expertise and prior knowledge remain key factors when choosing between ORA and FCS [21].

The third generation of enrichment techniques attempts to incorporate topological data on the underlying biological networks. This concept was applied early to genetic data through ScorePAGE [22] and is available in current tools like Pathway-Express [23]. For metabolomics data, MetaboAnalyst assigns each metabolic pathway a topological score accounting for the centrality of measured metabolites.

Pathway enrichment techniques face challenges, such as dealing with pathway crosstalk and overlap [14] or generating comprehensive outputs rather than pathway p-value lists [21]. Statistical tests that account for pathway crosstalk and overlap have been proposed for gene data [24, 25]. Although pathway analysis techniques constitute essential resources for metabolomics secondary analysis, the abstract and artificial borders between pathways may not faithfully reflect biological mechanisms [2]. This issue can be bypassed using sub-network analysis, a secondary analysis procedure to infer relevant biological modules under the condition of study [26] without being limited by pathway definitions. Sub-network analysis has also been applied to the canonical pathways to obtain enrichment in a sub-pathway scale for gene and protein data [27, 28]. Some methods, such as jActiveModules [29], define scores and attempt to find optimally scoring sub-networks. Likewise, diffusion kernels and random walk algorithms that score the nodes of a network, such as PageRank [30], have been applied to genetic data [31, 32] and metabolic networks [33].

The HotNet algorithm [31], applied to gene networks, computes pairwise influence measures from node *g*_*s*_ to node *g*_*i*_, by introducing a flow on *g*_*s*_ and allowing it to leave through all the nodes. The diffusion score of node *g*_*i*_, $f_{i}^{s}$, is interpreted as the influence *i*(*g*_*s*_, *g*_*i*_). A new undirected graph is built using the weights *w*(*g*_*j*_, *g*_*k*_) = min[*i*(*g*_*j*_, *g*_*k*_), *i*(*g*_*k*_, *g*_*j*_)], in which sub-networks encompassing a large number of gene mutations are sought. TieDIE [32] applies a similar concept, aiming to connect a source and a target gene set. Flow is introduced between the source and the target sets, giving rise to two diffusion processes that score all the nodes. The linking score of each node, defined as the minimum of its two diffusion scores, serves as a ranking to apply a global threshold and report the resulting sub-network.

Here we describe the development of an innovative methodology that combines the usefulness of pathway enrichment with the flexibility of sub-network analysis. Starting from summary metabolomics data, we apply a null diffusive process over a network-based representation of the KEGG database and derive a relevant sub-network. Besides offering an overview in the form of a list of affected pathways, we propose a novel sub-pathway representation at several molecular levels that justifies the reported pathways through additional biological entities (reactions, enzymes and KEGG modules) to identify candidates for further study. All of the reported entries, along with their annotations, are drawn in a heterogeneous network layout.

## Materials and methods

### Overview

**Fig 1:**
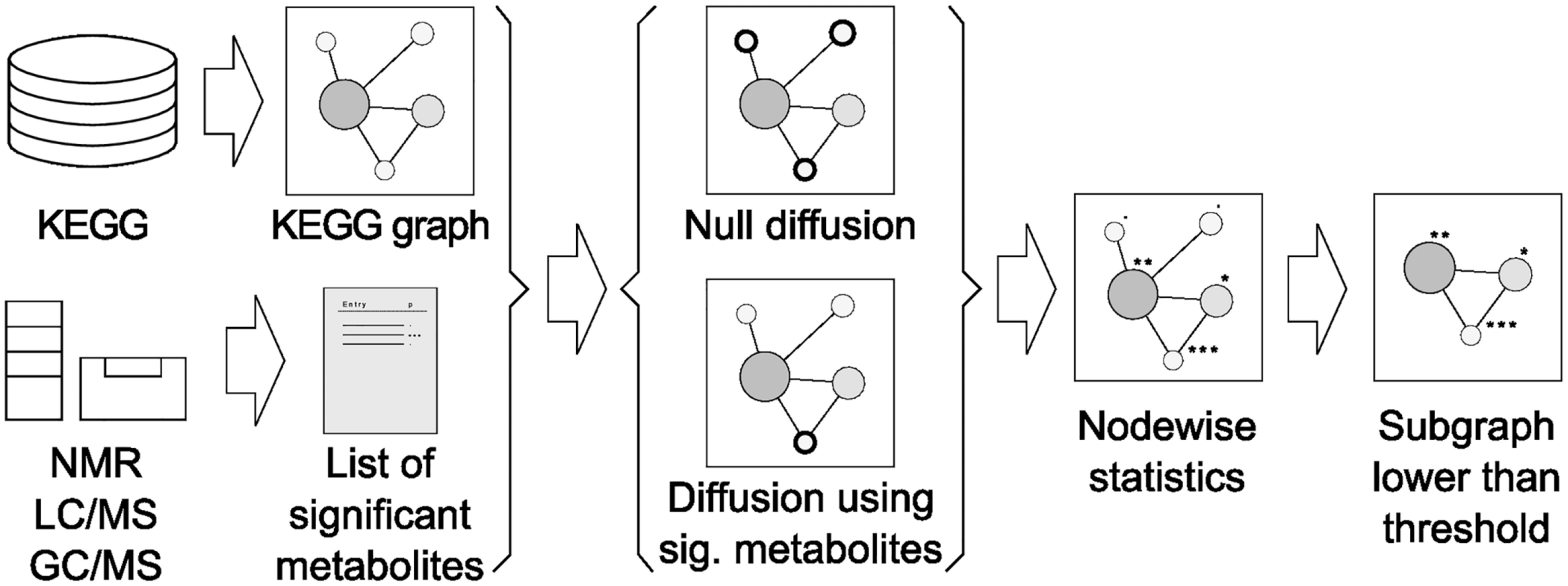
Workflow summary. Contextual knowledge is extracted from KEGG as a graph object while experimental data is introduced as a list of affected metabolites. A null diffusive model assesses, and reports in a subgraph, which part of the KEGG graph is relevant for the input metabolites.

An overall scheme of the proposed methodology is presented (Fig 1): on the one hand, we retrieve knowledge from KEGG as a graph object; on the other hand, the input to our algorithm is a list of significantly affected metabolites from an experimental study, obtained for example by applying a non-parametric Wilcoxon test to each metabolite’s abundance. Afterwards, the graph is regarded as a meshed object in which the nodes representing the affected metabolites introduce unitary flow. The resulting node scores are normalised using a null diffusive model, and the top scores define an interpretable relevant subgraph. All this work has been implemented in the R language [34] and the network algorithms rely on the igraph R package [35]. Our R code is under active development and available at https://github.com/b2slab/FELLA.

Contextual knowledge is depicted according to the KEGG database (Fig 1), through the following categories: compounds, reactions, enzymes, modules and pathways. This network is specific for Homo sapiens and its construction is detailed in S1 Appendix.

### Scoring algorithms

We derived scores for all the nodes through random walks on the KEGG graph, in order to assess their importance relative to the metabolites in the input. Performing random walks on the undirected graph is equivalent to running a diffusion process; specifically, we model heat diffusion. Conversely, if the graph is directed, the problem matches the PageRank algorithm for website ranking. Both the undirected and the directed versions are applied and referred to as diffusive processes (Fig 1).

**Fig 2:**
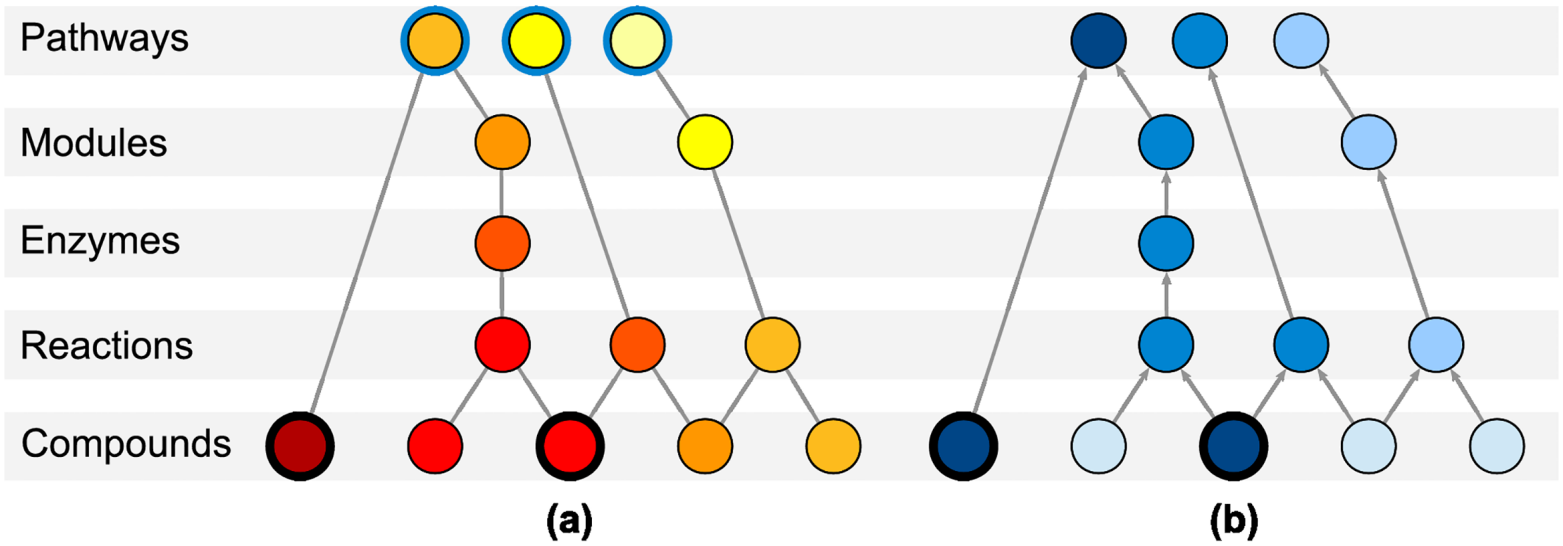
Nodes arrangement for (a) heat diffusion and (b) PageRank. The affected metabolites are highlighted with a black ring. For heat diffusion **(a)**, affected metabolites are forced to generate unitary flow. Every pathway is highlighted with a blue ring, representing its connection to a cool boundary node. In equilibrium, the highest temperature pathways (and nodes) will have the greatest heat flow, suggesting a relevant role in the experiment. For PageRank **(b)**, affected metabolites are the start of random walks. PageRank scores, represented by the intensity of the blue colour, will attain higher values in the frequently reached random walk nodes.

In the undirected graph case, using a heat diffusion model, we model the biological perturbation in the KEGG graph as heat flow that traverses our KEGG graph. It is important to emphasise that this heat diffusion approach is purely a knowledge propagation abstraction, in no way simulating heat diffusion on the actual biological entities. Heat is forced to flow from nodes corresponding to affected metabolites and through database annotations, leading to a score for each node in the KEGG graph: its stationary temperature (Eq 1). The rationale behind this approach is that nodes lying close to the affected metabolites, which are heat sources, will hold a higher stationary temperature. This can happen due to great proximity to a particular heat source or to overall closeness to multiple ones. In order to determine the temperatures, we apply the finite difference formulation [36] of the heat equation, using the explicit method, applied to a meshed object (Fig 2a) [37].

$$\begin{array}{c} T=-KI^{-1}\cdot G=R_{HD}\cdot G \end{array}$$ (1)

On the one hand, *KI* is the conductance matrix, where *KI* = *L* + *B*, *L* being the unnormalised graph Laplacian and *B* the diagonal adjacency matrix with *B*_*ii*_ = 1 if node *i* is a pathway and *B*_*ii*_ = 0 otherwise. The matrix *B* ensures that flow can leave the graph through pathways nodes. The matrix *R*_*HD*_ is defined as −*KI*^−1^, the linear mapping to compute the temperatures. On the other hand, *G* is the heat generation vector, whose entries *G*_*i*_ are unitary if *i* is an affected metabolite and 0 otherwise.

In our node arrangement (Fig 2a), the affected metabolites constantly introduce heat flow into the structure and only the nodes in the top level (metabolic pathways) are allowed to disperse it. Further details are available in S2 Appendix.

In the directed graph case, the PageRank scoring algorithm is a web model that assigns each website a score reflecting the number of incoming hyperlinks as well as the quality of their respective websites. The web surfer performs random walks on a directed graph, with an initial probability distribution over the nodes. In each step, the surfer resumes his random walk with probability *d* and restarts it with probability 1 − *d*, where *d* is the damping factor. If the surfer continues, he or she will choose an edge with a probability proportional to its weight. The default computation of PageRank scores is iterative for efficiency reasons, although a formula similar to (Eq 1) can be derived and will be used in the proposed methods. The damping factor is set to *d* = 0.85 as in the original publication.

The arrangement of nodes for the PageRank calculation is identical to the one for diffusion (Fig 2b), being edges directed towards the upper levels. Random walks start only at the affected metabolites and explore all the reachable nodes. Further details are available in S3 Appendix.

### Null models

**Fig 3:**
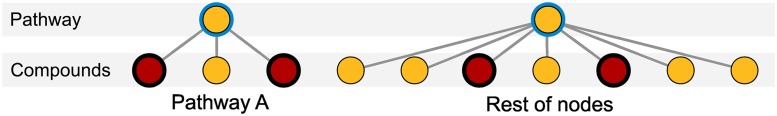
Toy example of an over-representation analysis of a hypothetical “pathway A” containing 3 metabolites out of a total of 10. The list to be enriched contains 4 metabolites, showing 2 hits in the pathway. The corresponding (Fisher’s exact test) over-representation can be understood as a diffusion process on the depicted network followed by a null model. The temperature of pathway A is always coincident with the number of hits in the pathway, implying that its null distribution is the hypergeometric distribution, to which a one-tailed temperature comparison is made.

The ranking of the network nodes is not achieved through raw scores, due to potential biases related to topological features. This is also the case in classical over-representation analysis, as it can be rephrased as a particular case of heat diffusion (Fig 3) where the observed statistic is the node temperature and its null distribution is the hypergeometric distribution. In view of this, our approach also includes a permutation analysis in the input, leading to a null distribution of scores for each node. Node scores are normalised using their null distributions and ranked, allowing a subgraph (Fig 1) to be extracted. Further details can be found in S4 Appendix.

The null model will be introduced in the heat diffusion scenario (the PageRank case is analogous). Let *n*_*in*_ be the number of compounds in the input. Then, exactly *n*_*in*_ different KEGG compounds are chosen at random following dependent Bernoulli distributions, so that *X*_*i*_ = 1 if *i* is chosen and *X*_*i*_ = 0 otherwise. Normalisation can be performed using (i) the theoretical mean and variance of the scores, which can be obtained from Eq 1, using the fact that, for the null model, *G* is a random vector *X* with known mean and covariance matrix:

$$\begin{array}{c} E\left(T_{null}\right)=R_{HD}\cdot E\left(X\right) \end{array}$$ (2)

$$\begin{array}{c} \Sigma \left(T_{null}\right)=R_{HD}\cdot \Sigma \left(X\right)\cdot R_{HD}^{T} \end{array}$$ (3)

The normalised score (z-score) of node *i* is defined in terms of the expected value $\mu_{i}=E{\left(T_{null}\right)}_{i}$ and standard deviation $\sigma_{i}=\sqrt{\Sigma {\left(T_{null}\right)}_{i,i}}$

$$\begin{array}{c} z_{i}=\frac{T_{i}-\mu_{i}}{\sigma_{i}} \end{array}$$ (4)

Then, nodes with the top *k* scores are kept and reported. Alternatively, scores can be normalised through (ii) Monte Carlo simulations with *n*_*perm*_ permutations, which provide an estimate of the probability *p*_*i*_ that the null distribution attains a score greater than or equal to the observed one. Estimation of *p*_*i*_ involves the empirical cumulative distribution function with a small correction [38], *r*_*i*_ being the number of permutations in which the null score of node *i* is greater or equal than *T*_*i*_:

$$\begin{array}{c} p_{i}=\frac{r_{i}+1}{n_{perm}+1} \end{array}$$ (5)

A consensus solution is derived from *n*_*vote*_ independent sets of Monte Carlo trials, each trial reporting the top *k* nodes. The consensus solution may therefore report a node count not exactly equal to *k*.

### NMR validation

The reported subgraphs contain entities other than pathways and compounds that can be useful for the researchers. Among these, the highlighted reactions have been partially validated by quantifying their distance to an independent second set of affected metabolites.

In order to analyse the reactions in the scope of a metabolic network, distances are computed on the unweighted, maximal connected subgraph containing all the compounds and reactions from the KEGG graph, referred to as the reaction-compound graph. The validation metric is the resistance distance, previously used in the chemical literature [39]. Under these settings, the reported reactions are compared to all the reactions that involve the input metabolites (their nearest neighbours) in terms of their resistance distance to the second set of metabolites.

### Evaluation with synthetic signals

In order to deploy an analysis of true and false positive pathway identifications, we opted to statistically characterize the pathway prioritisation induced by the diffusion scores. Artificial pathway signals have been generated to (a) find biases in the absence of a signal that might cause false positives, and to (b) quantify the ability to recover true positive pathways. The proposed methods are not directly compared to IMPaLA and MetaboAnalyst due to the lack of a batch analysis mode, but instead to their underlying distribution using Fisher’s exact test. Our Monte Carlo approaches have not been aggregated into consensus solutions. The performance metric is the pathway rank in the list ordered by a method, where $\frac{1}{n_{p}}$ is the best rank and 1 is the worst one, *n*_*p*_ being the number of pathways in the KEGG graph. Ranks in Fisher’s exact test are computed using the raw p-values, so that top ranked pathways correspond to lowest p-values. To compute the p-values, a metabolite is considered to belong to a pathway if it can be reached via the pathway in our directed KEGG graph (Fig 2).

In (a), noisy signals are generated and the ranks of all the pathways are calculated within signals. Then, the mean rank of a specific pathway *i* is computed across all the signals. This measure can reveal pathways that tend to have an extreme rank irrespective of the input.

In (b), a target pathway generates the signal and its rank is used as the metric of interest. Methods able to recover the signal will show low ranks in general terms.

### Description of the experimental data

Our method has been tested using data from a case-control experiment aimed at determining the function of an uncharacterised mitochondrial protein by silencing the gene using short hairpin RNAs (shRNA). Metabolites abundances were determined from five replicates of cell cultures expressing either control or experimental shRNA.

Metabolite measurements were performed by Metabolon platform (www.metabolon.com) using GC/MS (Thermo-Finnigan Trace DSQ single-quadrupole) and LC/MS (Waters ACQUITY UPLC and a Thermo-Finnigan LTQ-FT). The proprietary Metabolon analysis reported 168 quantified metabolites annotated in the KEGG database.

In addition, we have used NMR following the labelling of the same cells with [U-^13^C] glucose [40] to trace carbon atoms, in order to further validate the conclusions of our new method. The reported reactions are evaluated in terms of their resistance distance to the affected metabolites found by NMR.

### Description of the synthetic data

All the signals generate a list with fixed length *n*_*in*_ = 35 for each one of the *n*_*p*_ pathway nodes in the KEGG graph. Three sampling types have been defined—differences arise in the specification of how much more probable compounds in the target pathway are.

The first signal is a uniform sampling of *n*_*in*_ compounds that imitates noise: the probability of drawing a compound *j* within pathway *i*, *p*_*i*,*j*_, is *k*_*i*_ = 1 times more likely to be drawn than compounds outside the pathway, and thus does not depend on the pathway.

In the second signal, compounds belonging to pathway *i* are *k*_*i*_ = 10 times more likely to be drawn. Therefore, there are two different probability values: inside pathway and outside pathway. This sampling is affine to the assumptions in Fisher’s exact test from ORA.

As for the third signal, *p*_*i*,*j*_ is proportional to the quantity *R*_*HD*__*ij*_, which is greater in compounds close to the pathway. This takes into account the whole KEGG graph, thus being influenced by indirect connections and compound specificity.

## Results

### Input for the algorithms

After the curation step, our knowledge base graph contains 10,183 nodes and 31,539 edges. The nodes are stratified in 288 pathways, 178 modules, 1,149 enzymes, 4,699 reactions and 3,869 compounds. The degree distribution of its vertices follow a scale-free network model, where *P*(*k*)∼*k*^−*γ*^, with *γ* = 2.084 ∈ [2, 3], see S1 Appendix.

On the other hand, MS led to 168 quantified metabolites from KEGG. Two identifiers that each appeared twice have been dropped, as well as a KEGG drug, excluded from the KEGG compound category. The remaining 163 metabolites have been tested between both conditions, leading to 38 significant metabolites (two-tailed non-parametric Wilcoxon, FDR < 0.05), of which 33 have been mapped to our KEGG graph.

The 33 MS-derived compounds served as input for each of the proposed enrichment algorithms. Heat Diffusion (*HD*) and PageRank (*PR*) are followed by *norm* (z-score normalisation) or *sim* (Monte Carlo permutations). Normalised scores have been computed through the null models with *n*_*in*_ = 33, followed with subgraph selection with a desired number of nodes *k* = 250. For simulated methods, a consensus subgraph using *n*_*vote*_ = 9 runs of *n*_*perm*_ = 10,000 permutations each has been derived by majority vote on each node.

Regardless of the specific details, high diffusion scores are an indicator of overall closeness to the MS-derived metabolites and potential relevance in the condition being studied. This intuition, known as guilt-by-association, can be phrased in the context of heat diffusion: high temperatures are found close to the heat sources. Therefore, warm nodes are candidates for further study as they are easily reached through database annotations from the input metabolites.

### Null model impact

**Fig 4:**
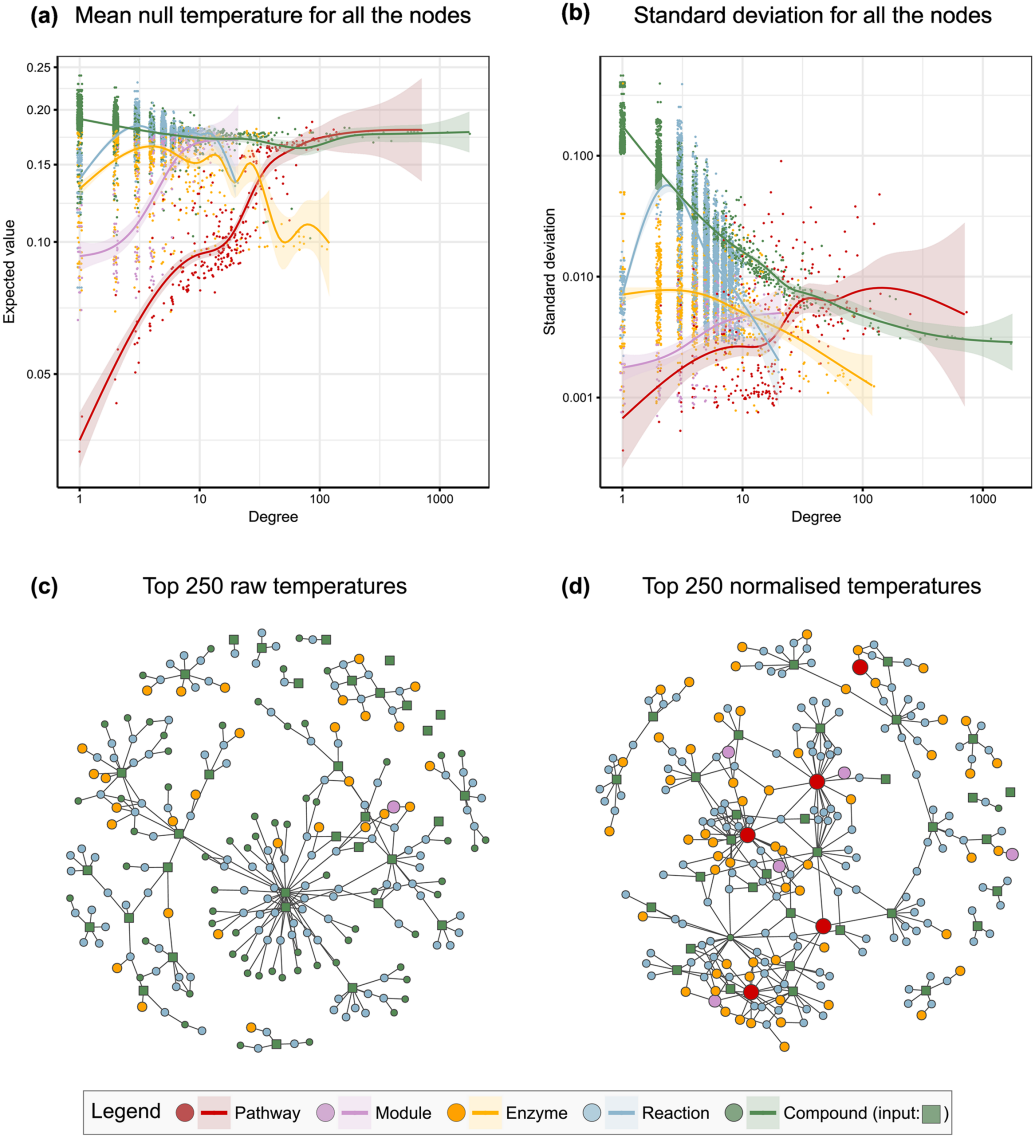
Expected value **(a)** and standard deviation **(b)** of the null temperatures, stratified by level—jitter applied for visual purposes and 0.95 confidence intervals computed by the default GAM models in ggplot2 R library [41]. Clear biases arise due to the node degree, a topological property of the nodes: the larger the pathway, the higher its mean value, and the more connected a compound is, the smaller its variance. If pathways are ranked by raw temperatures, a large pathway will have an undesired, consistent advantage over small ones and will be reported too often. The usage of z-scores **(d)** instead of raw temperatures **(c)** to select the top 250 nodes addresses these biases and highlights pathway and module nodes that were eclipsed by other compounds and reactions with higher mean null temperatures.

The impact of using the null model in HD and an overview of the random temperatures behaviour is described in Fig 4. The null model is closely related to the graph structure and node topology, quantified through the vertex degree. In Fig 4a, the mean temperatures show different trends for the five levels in the graph; in particular, there is an increase in the mean pathway temperature as the pathway becomes larger. This implies that, regardless of the input, larger pathways will generally show warmer temperatures and the results will be biased towards them. Likewise, the standard deviations of the null temperatures show level-specific changes (Fig 4b), with the compounds being the most affected entities—the higher the degree of the compound, the lower its standard deviation.

**Fig 5:**
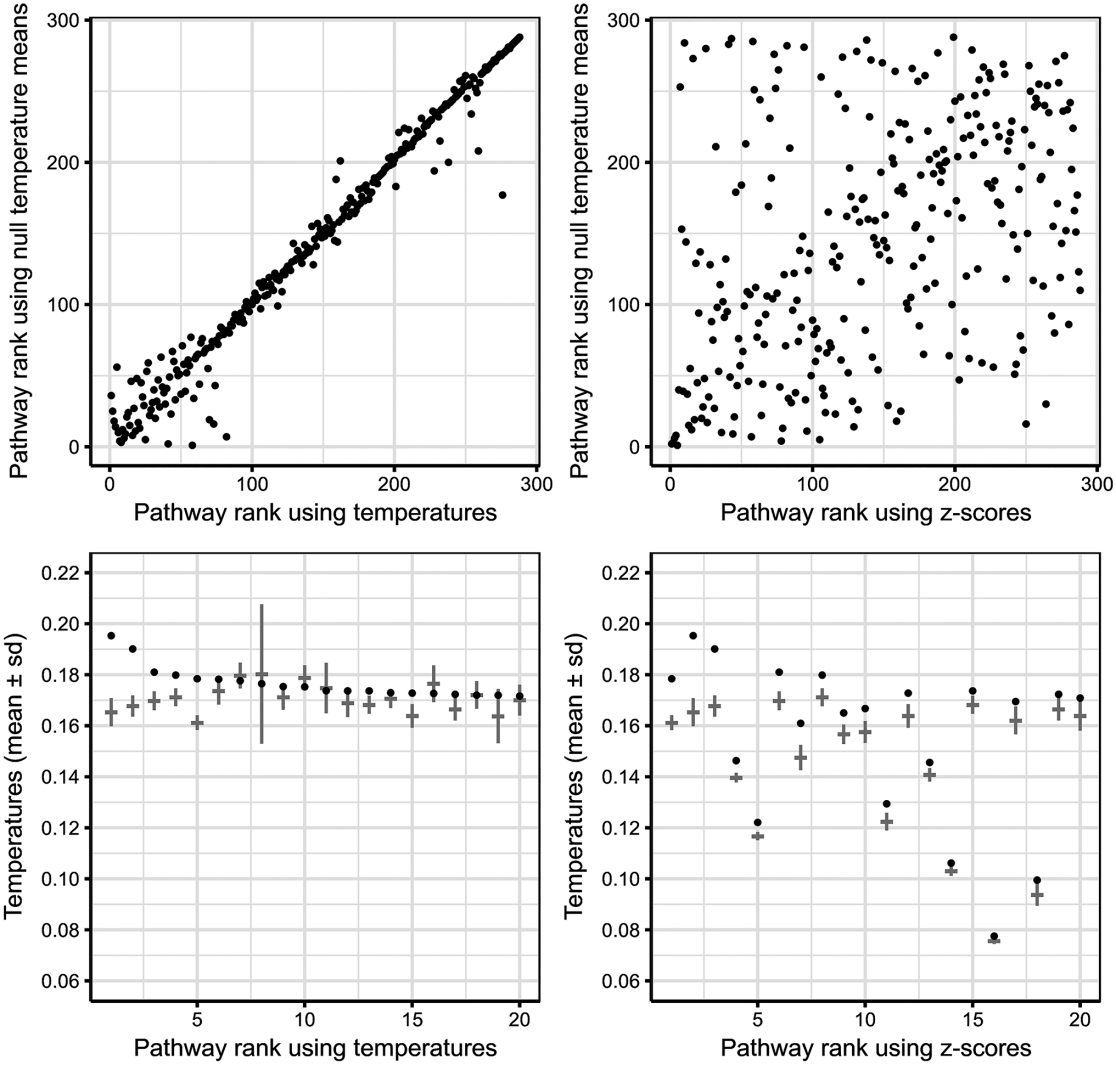
Ranking the 288 KEGG pathways—lower is best– using raw temperatures **(a)** biases the ranks towards pathways with higher mean null temperature, which in turn tend to be large pathways. Using the z-scores instead **(b)** breaks this clear dependence and avoids reporting pathways just because of their size. The top 20 pathways through raw temperatures **(c)**, depicted as black dots, include pathways that are even below their mean value, while the top 20 z-scores **(d)** suggest smaller pathways that were penalised by the aforementioned bias.

The usage of z-scores instead of raw temperatures has consequences in the highlighted nodes. Reporting the nodes with the top 250 raw temperatures does not reveal any pathway (Fig 4c), whereas five pathways lay among the top 250 z-scores (Fig 4d). Likewise, if only pathway nodes are considered, their ranking using raw temperatures is closely related to the ranking using the mean temperatures from the null model (Fig 5a), which is a property of the graph but not of the experimental data; using z-scores instead corrects this bias (Fig 5b). If the top 20 pathways are selected through their raw temperature, some of them are even below their mean null temperature (Fig 5c), whereas keeping the top 20 z-scores removes the bias towards larger pathways and suggests otherwise overlooked pathways (Fig 5d).

### Subgraph extraction

Four subgraphs have been extracted using the MS-derived compounds. The desired number of nodes *k* for each approach, together with the actual number of reported nodes and the number of KEGG pathways, are shown in Table 1. A connected component (CC) of an undirected graph is a maximal connected subgraph so that any two nodes in the subgraph are connected by a path. For the directed graphs, the weak CC definition is used, in which directed edges are considered as undirected when computing the CC. The number of nodes belonging to each solution subgraph, along with its largest CC and the number of CCs, are also reported. Additional details regarding the largest CC and number of CCs for other values of *k* can be found in S5 Appendix.

**Table 1:** Summary of the outputs.

Name	k	Pathways	Nodes	#CC	Largest CC
HD norm	250	hsa00250, hsa00270, hsa00480, hsa05230, hsa05231	250	8	206
HD sim	250	hsa00250, hsa00270, hsa00330, hsa00480, hsa05230, hsa05231	261	8	221
PR norm	250	hsa00250, hsa00270, hsa00480, hsa05231	250	9	187
PR sim	250	hsa00250, hsa00270, hsa00480, hsa05231	279	10	152

Summary of the outputs, using diffusion (HD) as well as PageRank (PR), and normalising the scores with Monte Carlo simulations (sim) or z-scores (norm). Monte Carlo simulations have been run 10,000 times per solution, and 9 solutions have been computed to build a consensus solution. Note that the desired number of nodes *k* is slightly different to the number of nodes actually reported in the Monte Carlo simulations. The last two columns contain the number of connected components (*CC*) and the number of nodes in the largest CC.

Defining the overlap coefficient between two solutions *G*_1_ and *G*_2_ as $\text{overlap}\left(G_{1},G_{2}\right)=\frac{|G_{1}\cap G_{2}|}{\min(|G_{1}|,|G_{2}|)}$, solutions tend to overlap despite their differences (Table 2). Regarding the stratification of the subgraphs in terms of KEGG categories, they follow a trend similar to the KEGG graph (S5 Appendix).

**Table 2:** Solutions overlap.

	HD norm	HD sim	PR norm	PR sim
HD norm	1.00	0.82	0.88	0.82
HD sim	0.82	1.00	0.77	0.83
PR norm	0.88	0.77	1.00	0.84
PR sim	0.82	0.83	0.84	1.00

Overlap coefficient statistics for HD and PR. The overlapping nature of solutions is a sign of consistency among approaches.

### Pathway analysis

Our methods are compared to IMPaLA and MetaboAnalyst to verify the concordance in terms of metabolic pathways. All the approaches have been compared using the example data from IMPaLA (S2 Table) and MetaboAnalyst (S3 Table), and they show consistent and compatible reports.

The results for our dataset are summarised in Table 3 and described in S1 Table, together with further details about the reports of the alternative tools. The metabolic pathways Alanine, aspartate and glutamate metabolism (hsa00250), Cysteine and methionine metabolism (hsa00270) and especially the Glutathione metabolism (hsa00480) recur in all of the approaches. Some of our solutions are more specific, suggesting the module Glutathione Biosynthesis (M00118) as well. Our null model takes pathway overlap and crosstalk into account and allows a visualisation of the pathway structure through the null diffusion correlation matrix (S4 Appendix).

**Table 3:** Reported pathways.

KEGG id	Pathway name	HD norm	HD sim	PR norm	PR sim	MA FCS	MA ORA	IMPaLA ORA
hsa00250	Alanine, aspartate and glutamate metabolism	+	+	+	+	+	+	-
hsa00270	Cysteine and methionine metabolism	+	+	+	+	+	+	+
hsa00480	Glutathione metabolism	+	+	+	+	+	+	+
hsa05230 (hsa00970)	Central carbon metabolism in cancer	+	+	-	-	*	-	+
hsa05231 (hsa00564)	Choline metabolism in cancer	+	+	+	+	*	-	-
hsa00260 (M00020)	Glycine, serine and threonine metabolism	*	*	-	-	+	-	-
hsa00330 (M00133)	Arginine and proline metabolism	*	+	-	-	+	-	+
hsa00510 (M00073)	N-Glycan biosynthesis	-	-	*	*	-	-	-

Pathways reported by our methods. ‘+’ means a hit for the term reported in the KEGG id column, ‘*’ stands for a hit of the closely related term in parenthesis in the same column and ‘-’ states no hit. Our 4 solutions are compared to MetaboAnalyst (MA), using ORA and FCS, and IMPaLA using ORA. Pathways hsa00250, hsa00270 and hsa00480 are repeatedly reported by all the methodologies. Pathways hsa05230 and hsa05231 are reported by some of our methods, while alternative approaches find some close (*) and exact (+) matches. In some cases, instead of reporting a whole pathway, only specific modules within it are reported as relevant; this is the case of M00133 and M00073. Furthermore, module M00073 does not contain any compounds, being out of the scope of MetaboAnalyst and IMPaLA, but is reported by one of our methods due to the presence of other indirect relationships through enzymes in the graph.

**Fig 6:**
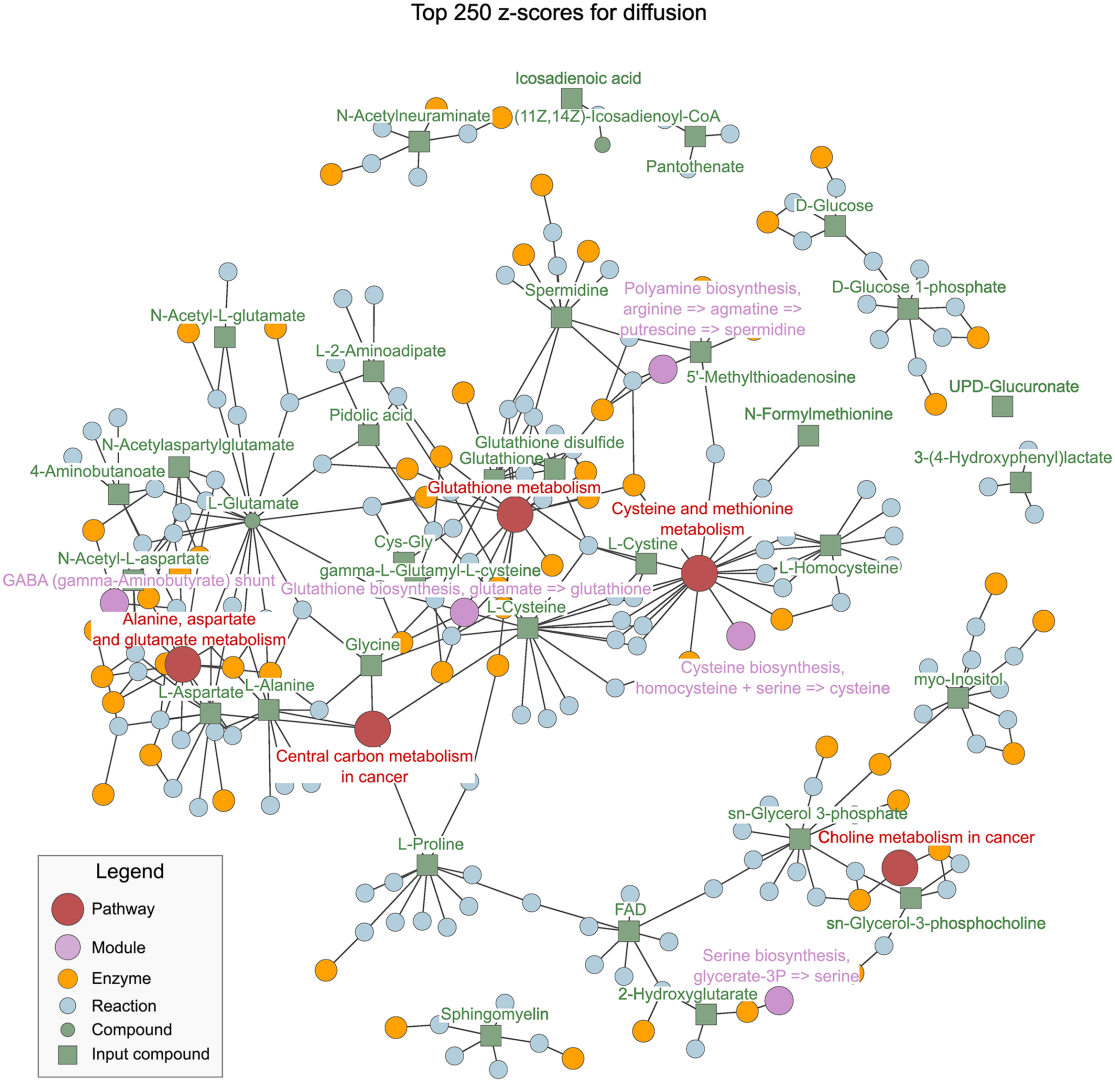
Subgraph reported through HD norm, the names of reactions and enzymes have been omitted for clarity. Compounds are green, reactions are blue, enzymes are orange, modules are purple and pathways are red. The compounds in the input are highlighted as green squares to ease the tracing of the biological perturbation up to the pathways. The presence of reactions and enzymes that link pathways in this subgraph might suggest relevant entities by which affected pathways crosstalk. All the reported pathways and modules lie in a large CC, as well as a newly proposed metabolite (L-Glutamate).

The subgraph resulting from applying HD sim (Fig 6) inherits the scale-free structure from the whole graph and enrols the three recurrently reported pathways in the same connected component: hsa00250, hsa00270 and hsa00480. The biological perturbation stemming from the MS-derived compounds can be tracked in terms of reactions, enzymes and modules, up to the relevant pathways.

**Fig 7:**
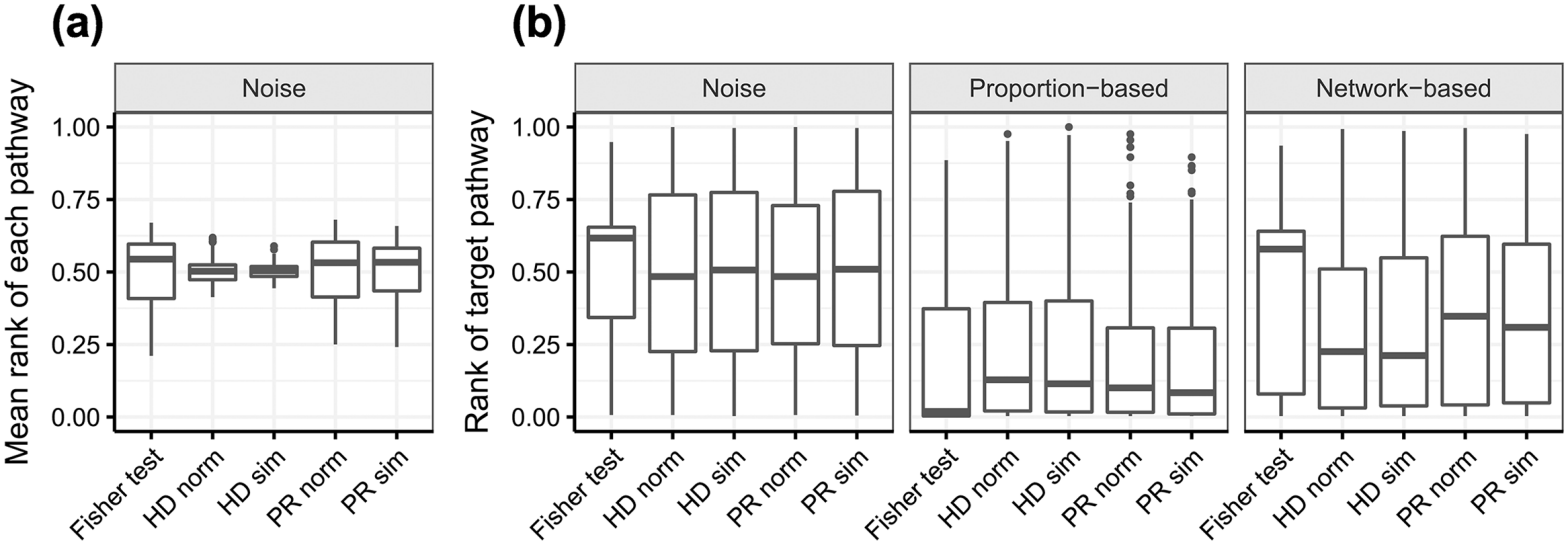
Synthetic signals evaluation using the pathway rank as a metric to assess orderings. Lowest ranks correspond to best ranked pathways. The proposed methodology is compared to ORA, represented by Fisher’s exact test. **(a)** 288 noisy signals have been generated, and every pathway has been ranked in each of the 288 runs. Data points for a given methodology are the mean rank of each pathway, giving 288 data points per box. **(b)** 288 signals with a target pathway have been generated, in three scenarios: pure noise, proportion-based sampling and network-based sampling. Each box contains the rank of the target pathway, leading to 288 data points per box.

On the other hand, results on the recovery of synthetic signals can be found in Fig 7. In (a) absence of signal, HD ranks pathways with a mean rank close to 0.5, and only a few are biased to the top or the bottom of the list. Mean ranks in Fisher’s exact test and PR are also centered around 0.5, but have more dispersion. In (b) the presence of a target pathway, three sampling schemes have been explored. In (1) the signal is actually noise and the target pathway is a decoy. The rank of the target pathway for HD and PR is uniformly spread in [0, 1], whereas Fisher’s exact test shows some asymmetry in the rank distribution. In (2), the sampling probability depends on the presence or absence of the metabolite in the pathway. Fisher’s exact test outperforms HD and PR as the median rank of the target pathway is closer to 0, as expected by its optimality. However, in (3), the sampling probability is network-based and HD outperforms PR, which in turn outperforms Fisher’s exact test. Differences between sim (Monte Carlo trials) and norm (parametric approach) are subtle.

### NMR analysis

**Fig 8:**
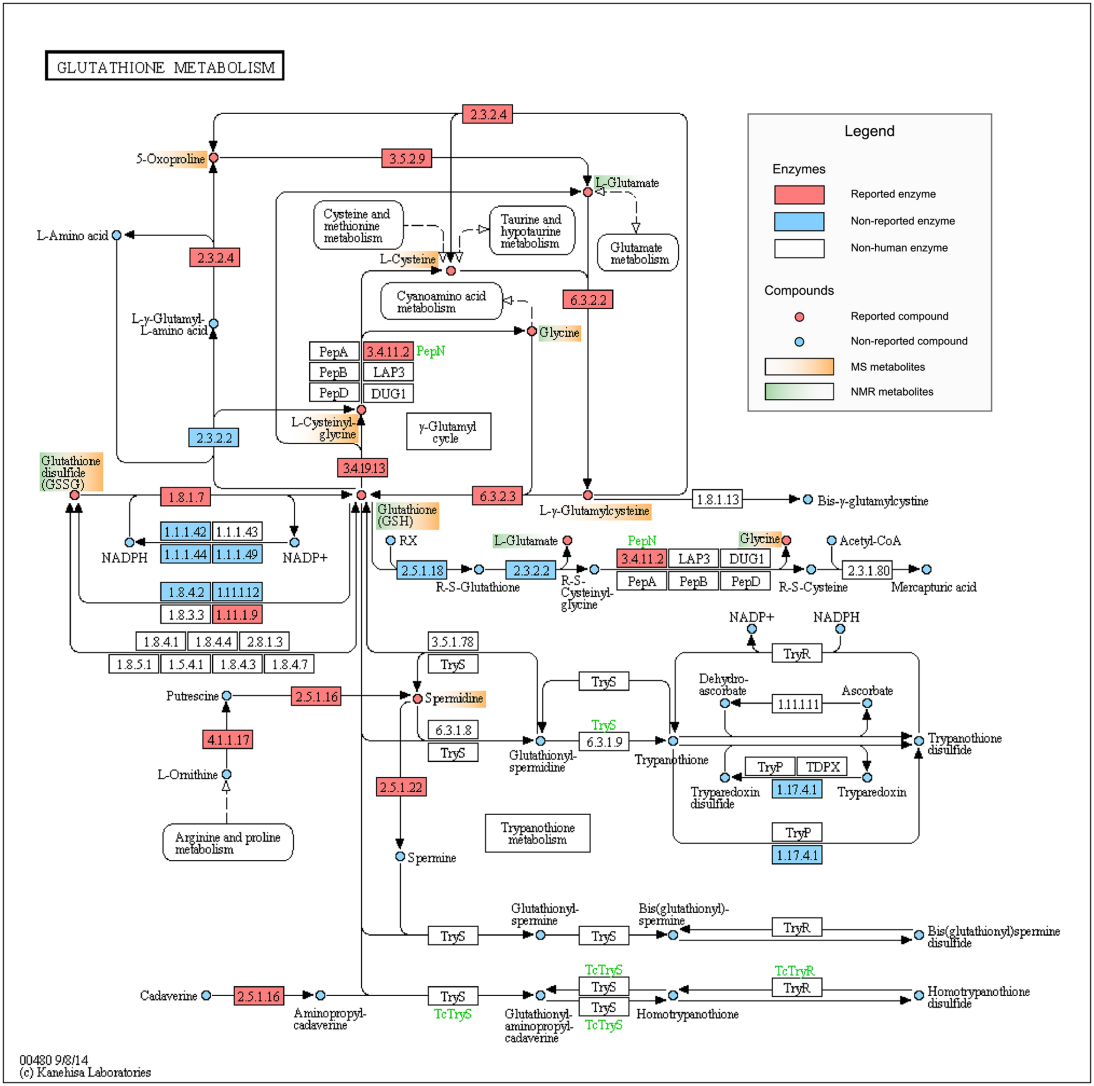
KEGG representation of the Glutathione metabolism (hsa00480). KEGG compounds found affected through MS (orange) and NMR (blue) are pinpointed in the figure. Additionally, enzymes and compounds reported by HD norm are depicted in red. Our approach provides a criterion for highlighting a pathway together with the entities it contains, for example its reported enzymes, to build a sub-pathway representation richer than the classical methods that rely solely on pathways and compounds. Reprinted from www.genome.jp under a CC BY license, with permission from Kanehisha Laboratories, original copyright 2014.

NMR carbon tracking revealed 13 isotopically enriched metabolites from ^13^C-glucose, showing differential fractional enrichment between case-control, of which 5 had already been found through MS; some of these metabolites can be seen in Fig 8 in the context of the Glutathione metabolism. Our solutions are assessed in terms of the resistance distance from the reported reactions to the remaining 8 metabolites. The smaller the overall distance of a solution, the more related its nodes are to the 8 metabolites proven affected by NMR. The resistance distances have been computed on the reaction-compound graph, which is the largest CC of the subgraph that contains all the reactions and compounds in the KEGG graph.

The reactions suggested in our subgraphs show lower resistance distances to the 8 NMR-derived metabolites than the totality of reactions in the reaction-compound graph (Table 4). Furthermore, they are also lower than the resistance distances from the neighbouring reactions of the MS-derived metabolites to the 8 NMR metabolites (FDR < 0.01).

**Table 4:** Distance to NMR metabolites.

Method	Graph order	C00299	C00122	C00116	C00105	C00020	C00581	C00300	C00025
Reaction-compound graph	4539[8008]	0.56(0.62)	0.56(0.62)	0.57(0.62)	0.54(0.62)	0.47(0.62)	0.93(0.62)	0.82(0.62)	0.47(0.62)
First neighbours	414[447]	0.42(0.12)	0.43(0.12)	0.44(0.12)	0.40(0.12)	0.33(0.12)	0.79(0.12)	0.68(0.12)	0.33(0.12)
HD norm	147[250]	0.39(0.10)	0.39(0.10)	0.40(0.10)	0.37(0.10)	0.30(0.10)	0.76(0.10)	0.65(0.10)	0.30(0.10)
HD sim	148[261]	0.39(0.09)	0.39(0.09)	0.40(0.10)	0.37(0.09)	0.30(0.09)	0.76(0.09)	0.65(0.09)	0.30(0.09)
PR norm	143[250]	0.39(0.10)	0.39(0.10)	0.40(0.10)	0.37(0.10)	0.30(0.10)	0.75(0.10)	0.65(0.10)	0.30(0.10)
PR sim	172[279]	0.40(0.12)	0.41(0.12)	0.42(0.12)	0.38(0.12)	0.31(0.12)	0.77(0.12)	0.66(0.12)	0.31(0.12)

Mean resistance distance between the reactions reported in our solutions and each compound reported using NMR, with their standard deviations in parentheses. For each subgraph of KEGG graph, the number of reactions and the total number of nodes (in square brackets) are displayed. The reaction-compound subgraph contains the largest connected component having all the reactions and compounds in the KEGG graph. The first neighbours subgraph contains the MS-derived metabolites and all the reactions in which they participate. Resistance distances are computed on the reaction-compound graph. For every NMR-derived metabolite, there is a significant difference in resistance distances between the reactions proposed in our solutions and the reactions involving any of the MS-derived metabolite (one-sided Wilcoxon test, FDR < 0.01 for the 32 possible comparisons: 8 NMR metabolites, tests of 4 solutions against the first neighbours reactions). This implies that the reported reactions are closer to the NMR-derived compounds than the bulk of neighbouring reactions.

## Discussion

Our approach for enriching summary metabolomics data, Fig 1, is based on diffusion processes over a graph drawn from several KEGG categories (Fig 2). KEGG is the database of choice due to its level of curation and structure, which eases the graph representation. Specifically, the definition of KEGG categories naturally allows a hierarchical arrangement of levels. Our graph design is enhanced by the compound-reaction-enzyme-gene networks built by MetScape (S1 Appendix), and the inclusion of modules and pathways in our arrangement allows a comprehensive picture of the affected biology.

The graph contains all the KEGG compounds and the subset of affected metabolites forced to diffuse inside it (Fig 2). The closer a node is to the affected compounds, the higher its score becomes. Likewise, the top scoring candidates naturally involve higher flow and become relevant in the flow discharge from the graph. Because our KEGG graph is conceived and curated in a bottom-up manner, diffusion is expected to follow that trend too: the perturbation in the lowest level will diffuse to the upper levels to exit the graph. Ideally, a relevant subgraph found through this diffusion (Fig 6) would inherit the stratification of the KEGG graph, thus allowing the extrapolation of knowledge in terms of compounds to the rest of categories. This allows holistic picturing of pathways of interest, such as Glutathione metabolism (Fig 8) and importantly, it relates affected pathways through reactions, enzymes and compounds.

The mathematical formulation of the heat diffusion stationary temperatures is equivalent to the scores in HotNet and TieDIE, with ad-hoc boundary conditions (Fig 2). Conversely, our settings for PageRank force upwards diffusion and allow exit from every node through the damping factor. Node selection for HotNet follows a combinatorial model, whereas TieDIE applies a unique threshold for all the scores, which in turn come from two diffusive processes. In our case, selection is achieved through a unique diffusion followed by a null model that normalises the scores. Comparing raw scores between nodes can lead to biases related to the node level and topology (Fig 4a and 4b), pathway nodes clearly being affected by their degree and, in addition, overshadowed by other compounds and reactions with higher mean null temperatures. Without further action, the temperatures of larger pathways are systematically warmer regardless of the input, thus biasing all the results and any biological interpretation. Instead, our concept of a high score for a given node relies on comparing its score to its null distribution, treating each node according to its own topological features (Fig 1).

This is consistent with the pathway over-representation analysis, as the latter can be posed as a very simple diffusion problem that needs the null model to translate the observed statistics into p-values that are comparable across pathways (Fig 3). Ranking pathways by the number of hits and ignoring the null model would bias the results towards larger pathways, which is also what happens in our diffusion approach if raw temperatures are used (Fig 5a and 5b).

Finally, we extract four subgraphs by considering the top *k* scores for HD norm, HD sim, PR norm and PR sim. Spurious highlighted nodes are expected to appear as isolated or having very small CCs, similar to random selection of nodes in a sparse graph, whereas strong biological perturbations yield larger CCs. Therefore, the large CCs reported in the four subgraphs (Table 1) are natural goodness-of-solution indicators.

Analysing the two statistical approaches, we suggest both deterministic parametric techniques and stochastic non-parametric ones. Computing a z-score is simple and fast, giving insights into how high a score is in terms of standard deviations from the mean value. On the other hand, Monte Carlo trials can show some variability between solutions, so an ensemble approach can address this, while providing confidence measures for each reported node. Conversely, several quantiles can be estimated and stored if the graph is unchanged for further analyses, which is reasonable for a given KEGG database release.

Regarding time and memory complexity, the complete analysis of the database requires a one-off computation the inverse of the conductance matrix of the graph, which is feasible in our scenario and already pre-computed for our public package. The cost of the Monte Carlo trials is benchmarked in S5 Appendix. Comparing both random walk approaches, we observe a tendency to report larger CCs through heat diffusion (Table 1), because it can propose new compounds in the solution that connect otherwise disjoint CCs. This is not the case for PageRank, as forcing the diffusion upwards excludes other compounds from being visited by the random walks. As expected, all the approaches tend to report the metabolites that were specified in the input, although the z-scores can be more restrictive when suggesting new compounds in heat diffusion, possibly due to their high variance. Despite the differences between scoring methods and statistical approximations, solutions show a consistency because of their high overlap (Table 2). Furthermore, reporting subgraphs with a stratification similar to the KEGG graph (S5 Appendix) indicates perturbation traceability and allows inference on various KEGG categories by measuring only compounds.

As a pathway enrichment method, our procedure shows results consistent with the state of the art. Artificial signals have been generated to discover biases in particular pathways and assess the goodness of the rankings produced by the methods. In (a) the absence of signal, the mean rank of a pathway is expected to be uniform on [0, 1] and have a mean value of 0.5. If the mean value is closer to 0, the pathway might be systematically favoured in any analysis and could become a recurrent false positive. HD shows small deviations from 0.5 in the mean rank of the 288 pathways in the KEGG graph while PR and Fisher’s exact test show more dispersion. This may be due to the discrete nature of Fisher’s exact test, which is partly inherited by PR as it only allows upwards propagation. In (b) the presence of signal, a target pathway generates the signal and is ranked in the prioritisation of each method. In the first sampling scheme, the target pathway is actually a decoy and is expected to be ranked uniformly on [0, 1]. This is the case for HD and PR, but Fisher’s exact test shows an asymmetrical distribution, probably a consequence of pathways tied at 0 hits. If the sampling strategy is affine to Fisher’s exact test alternative hypothesis, this test has an edge over HD and PR in terms of discovering the true positive. Conversely, if the sampling is network-based, HD and PR perform better, as the binary nature of Fisher’s exact test cannot account for metabolites close to, but not inside of, a target pathway. This sampling generates signals that are harder to recover because of the network topology: crosstalk effects are present and unspecific metabolites divide their contribution over all the pathways to which they belong. This implies that, focusing on the pathway ranking problem, the optimal choice between Fisher’s exact test and HR or PR depends on the network influence in the generative model of the data.

An added value of our approach is in providing further details about the reported pathways, together with more specificity due to the presence of KEGG modules. Our results offer sub-pathway resolution and, unlike other sub-pathway focused tools, details at several molecular levels between the metabolites and the pathways. Entities like enzymes or metabolites that appear relevant and shared among pathways can give insights of pathway overlap and crosstalk that is specific to the condition under study. Our pathway hits are consistent with the current techniques, both using list format and abundance data (Table 3). The same tendency is observed when benchmarking with IMPaLA and MetaboAnalyst example data, details in S2 and S3 Tables. However, the nature of our scores takes into account pathway overlap, which is not the case for IMPaLA (ORA) and MetaboAnalyst (ORA and MSEA).

Our prior studies [42] suggest that the Glutathione metabolism (Fig 8) is of particular interest and it is consistently pinpointed by the enrichment methods. Its study is illustrative of the workings of our methodology: nodes surrounding the input metabolites support warmer temperatures and hence the proposed enzymes within the pathway are close to the MS-derived metabolites. The suggestion of these enzymes gives a richer view within the pathway and can help generate new biological hypotheses. This context also depicts L-glutamate, an extra metabolite suggested by the method, which is surrounded by MS-derived metabolites and also found through NMR.

The lack of a gold standard procedure and a reference benchmark dataset with known biology for pathway enrichment [14, 21] encouraged the analysis of metabolic changes using isotopic labelling and NMR. The novelty of our tool includes the generation of a comprehensive subgraph that contains more than pathways and compounds—consequently we also partially validate the reactions that appear in the subgraph. The definition of performance is not straightforward, given the lack of means to prove that a node (compound, reaction) is not affected, so the usual quality measures (false positives, true negatives) are not applicable. Results show that our reported reactions have lower resistance distances to the 8 metabolites found by NMR than all the reactions involving any of the MS-derived metabolites (Table 4). The choice of resistance distance as a validation metric is motivated by the presence of hubs in the metabolic network that affect the usual shortest paths metrics, meaning that connections through very specific metabolic reactions are masked by very general reactions involving hubs like adenosine triphosphate (ATP). As resistance distance takes into account the whole graph structure, and specifically the presence of multiple shortest paths, it is more informative than shortest paths distance.

## Conclusions

We propose a secondary analysis methodology for summary metabolomics data that combines pathway enrichment and sub-network analysis. Instead of reporting a list of pathways, we build meaningful sub-pathway representations of the biology at several molecular levels, derived through a null diffusive process on a curated graph object built from the KEGG database. This approach accounts for pathway over-representation, topology and crosstalk. Nodes reported as relevant are drawn in a comprehensive heterogeneous network that contains not only pathways and compounds, but also enzymes, reactions and KEGG modules. This richer biological context adds value to the top pathway hits by suggesting possible paths through which affected compounds translate into dysregulated pathways.

The proposed methodology has been tested and assessed in a case-control study, where the suggested pathways are consistent with alternative pathway enrichment techniques and the reported reactions have been partially validated through NMR-based tracking of glucose carbon. Our analysis suggests that the Glutathione metabolism is one of the most affected pathways. Glutathione is critical for the suppression of reactive oxygen species and this result is consistent with our preliminary observations that these cells exhibit higher levels of mitochondrial reactive oxygen species. Tests on simulated data suggest that our methodology can benefit from pathway signals whose generative model is network-based. These results support the potential of our novel methods for aiding in the interpretation of complex metabolomics datasets.

## Supporting information

S1 Table Experimental data results. Reported subgraphs and pathway analysis using IMPaLA and MetaboAnalyst on the experimental dataset.
(XLSX)

S2 Table IMPaLA example data. Reported pathways for the IMPaLA example data using top 250 z-scores in heat diffusion, IMPaLA and MetaboAnalyst.
(XLSX)

S3 Table MetaboAnalyst example data. Reported pathways for the MetaboAnalyst example data using top 250 z-scores in heat diffusion, IMPaLA and MetaboAnalyst.
(XLSX)

S1 Appendix Graph structure and curation. Details on how to generate and curate the KEGG graph.
(PDF)

S2 Appendix Heat diffusion process. Formulation of the heat diffusion scoring method.
(PDF)

S3 Appendix PageRank. Formulation of the PageRank web ranking algorithm.
(PDF)

S4 Appendix Null models. Definition of the null models and visualisation of the pathway correlation matrix.
(PDF)

S5 Appendix Details on reported solutions. Solution stratification, CC evolution, computational cost of Monte Carlo permutations and damping factor influence.
(PDF)
