# Supplementary material for: Null diffusion-based enrichment for metabolomics data
Source: PLoS One. 2017 Dec 6;12(12):e0189012. doi: 10.1371/journal.pone.0189012 (PMC5718512; doi:10.1371/journal.pone.0189012)
Supplement: S1 Appendix — Details on how to generate and curate the KEGG graph. (PDF) [file pone.0189012.s004.pdf]

Appendix S1 - Graph structure and curation

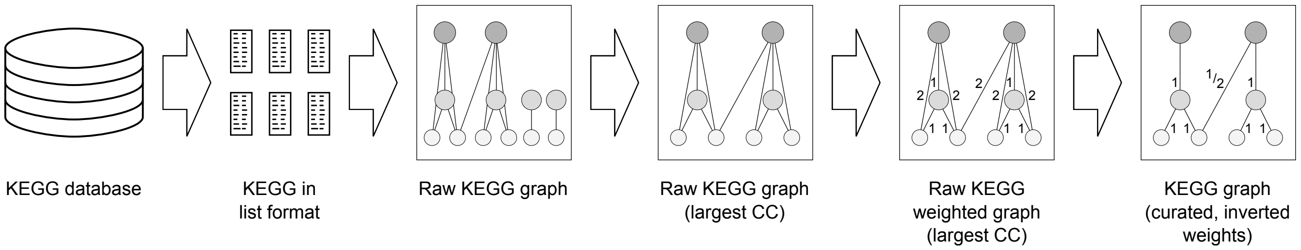

Figure A: Procedure to obtain the KEGG graph. The KEGG database is read as a collection of lists that contain the annotations. The raw KEGG graph is built through these annotations, where the vertices are KEGG entries from categories in Fig. Ba. We only work with the largest CC of the raw KEGG graph, to which weights are assigned, enabling the curation step that gives place to the KEGG graph. Note that the weights in the definitive KEGG graph are the inverse of the former dissimilarity weights, to be consistent with the diffusive methods.

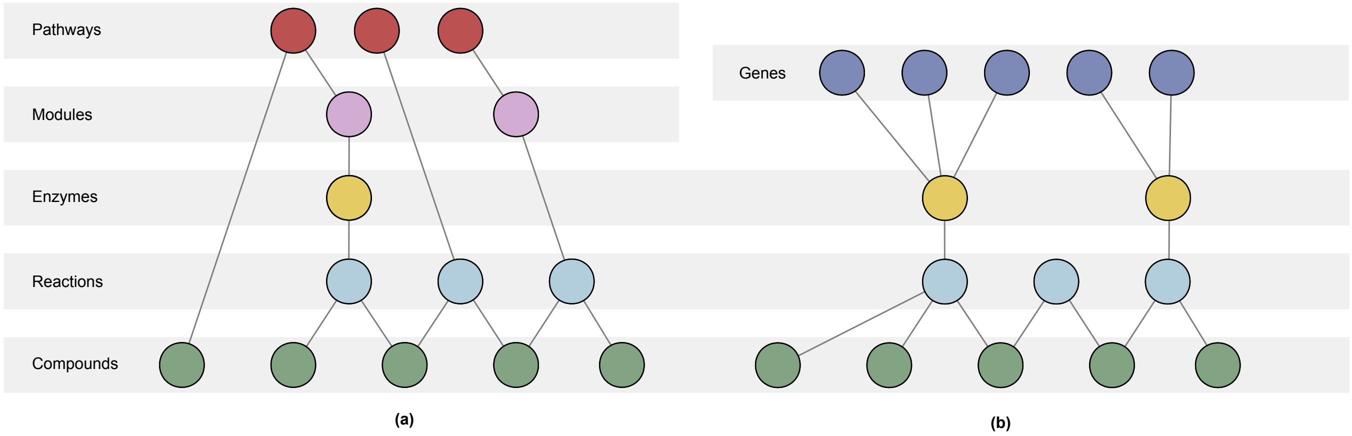

Figure B: **(a)** Structure of our KEGG graph. Each entry belongs to a level, ranging from 1 (pathways) to 5 (compounds). **(b)** MetScape concept of compound-reaction-enzyme-gene network. Their construction is similar in the three lowest levels, while in the upper level they include KEGG genes.

The first step to depict current knowledge is to build a graph object from KEGG that enables data enrichment (Fig. A). The KEGG graph contains various categories (Fig. Ba) and keeps similarities with the networks built through MetScape (Karnovsky et al., 2012), see (Fig. Bb), although our structure is conceived to include biological pathways and modules to obtain a pathway enrichment procedure. The lists relating these categories can be retrieved through the KEGGREST package (Tenenbaum, 2016).

In order to restrict our KEGG graph to nodes related to Homo sapiens, only human pathways and modules were considered. Pathway hsa01100 (Metabolic pathways) was discarded for being too general. Enzymes were included only if at least one human gene was related to them, as enzyme-module and enzyme-pathway connections were inferred through genes. Reactions and compounds were drawn only if they belonged to a human pathway or module. Finally, for completeness purposes, any reactant or product of the already kept reactions was added. These steps resulted in the raw KEGG graph (Fig. A).

We have conceived a curation algorithm that assigns edge weights and removes redundant edges from the graph. The requirements for the graph that enable the curation and the diffusion processes are: **(i)** the chosen categories allow a hierarchical arrangement, **(ii)** none of the links relates nodes belonging to the same category and **(iii)** affected nodes lie only on the bottom level (lowest category).

In the first place, our five KEGG categories conform a hierarchy, from top to bottom: biological pathway, module, enzyme, reaction and compound. This choice mimics the transition from the smaller parts (compounds) to the larger units (pathways) and facilitates the tracking of the biological perturbation, suggesting paths and entities by which the affected compounds translate into altered pathways. In the second place, KEGG does not contain any link between entries within the same category.

After building the unweighted graph from KEGG annotations and working with its largest CC, we begin the curation by proposing edge weights (Fig. A) that reflect the specificity of the link between the two entries i and j within the hierarchy, as described in equation (1):

$$w_{ij} = w_{ji} = \begin{cases} |l_i - l_j| \text{ if } i \text{ and } j \text{ are linked through an edge} \\ \infty \text{ otherwise (equivalently, not adjacent vertices)} \end{cases} \quad (1)$$

In equation (1), l_i stands for the level of node i; note that the specified requirements ensure that l_i is defined for each node (hierarchical structure) and that $w_{ij} \neq 0$ (no edges between nodes within the same level). For instance, an edge between a compound and a reaction weights 1, meaning that it describes a close relationship in metabolic terms. Instead, if the link involves a compound and a pathway this weight becomes 4, meaning the lack of known intermediate implications involving reactions, enzymes and modules.

The next step in the curation process discards any edge that can be explained using more informative edges (Fig. A), therefore avoiding any data loss. Specifically, any triangle in the graph is removed by dropping the edge with the largest weight. For example, a link between a compound and a pathway will drop if there are known intermediate levels that explain this connection.

The algorithm to achieve this from the original weighted graph $G = (V, E)$ of order n and size m is the following. Note that the algorithm is still valid in the presence of multi-edges (edges that are incident to the same pair of vertices), but as a proof of concept we assume that the graph does not contain them.

1. Sort the edges in E with increasing w_{ij}: $L = (e_{(1)}, \ldots, e_{(m)})$. The criterion to break ties is irrelevant.
2. Initialise a graph $G_{new} = (V_{new}, E_{new}) = (V, \varnothing)$ with the same node set as the original graph, but with no edges
3. For each edge e_{ij} in L, which links vertices i and j in G, add e_{ij} to G_{new} only if $d_{G_{new}}(i, j) > w_{ij}$.
4. Return G_{new}

In other words, only edges that contribute with new data in the biological graph are added. Distances must use the weights provided by w_{ij}. If an edge e_{ij} is discarded, that means that there is already a connection between i and j with the same level of detail, and because of the construction of G_{new} this connection is through two or more edges, all of them having strictly less weight than e_{ij}. Hence, e_{ij} is redundant in that situation. A small example is shown (Fig. C) to justify the curation process.

After the curation process, we obtain the KEGG graph (Fig. A). The final weights are inverted to be consistent with the graph Laplacian matrix and the diffusive methods. KEGG graph contains a total of 10,183 nodes and 31,539 edges. The nodes are stratified in 288 pathways, 178 modules, 1,149 enzymes, 4,699 reactions and 3,869 compounds. The degree distribution (Fig. D) follows a scale-free model.

The third requisite about the graph (the measured nodes should lie on the lower level) ensures that the boundary setup is meaningful and it eases the traceability of the biological perturbation, which follows a bottom-up tendency. Introducing flow on intermediate levels can nullify the structure inheritance from the whole graph when selecting the subnetwork, thus undermining the quality of the resulting biological interpretation.

The application of our diffusion processes with their null models is aimed at reporting a relevant subgraph of our KEGG graph. This subgraph can be examined through the order (amount of nodes) and amount of connected components (CC), an indicator of its structure and quality. A large CC is likely to give a global explanation in terms of all the levels in the graph while several small CCs will only highlight very specific relationships between small sets of nodes.

References

Karnovsky, A., Weymouth, T. E., Hull, T., Tarcea, V. G., Scardoni, G., Laudanna, C., Sartor, M. A., Stringer, K. A., Jagadish, H. V., Burant, C. F., Athey, B. D., and Omenn, G. S. (2012). Metscape 2 bioinformatics tool for the analysis and visualization of metabolomics and gene expression data. *Bioinformatics*, 28(3):373–380.

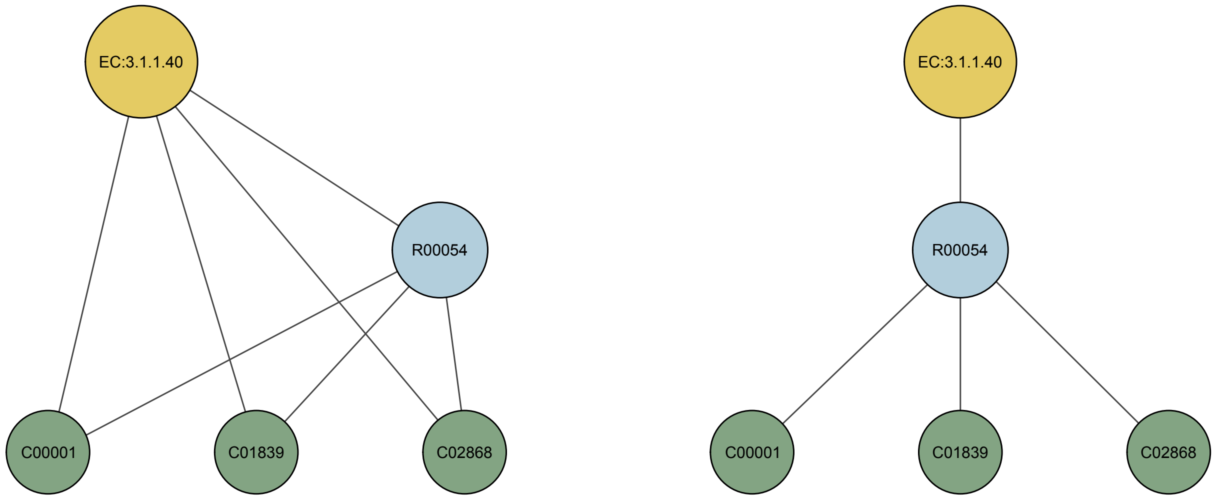

Figure C: Example of the curation process applied to a small subgraph from KEGG graph. The graph in the left contains all the original edges. Likewise, the graph in the right contains a neater explanation of the biology: three compounds that participate in a reaction, catalysed by one enzyme. We capture the essence of the data through 4 edges instead of 7, while easing the posterior visual interpretation.

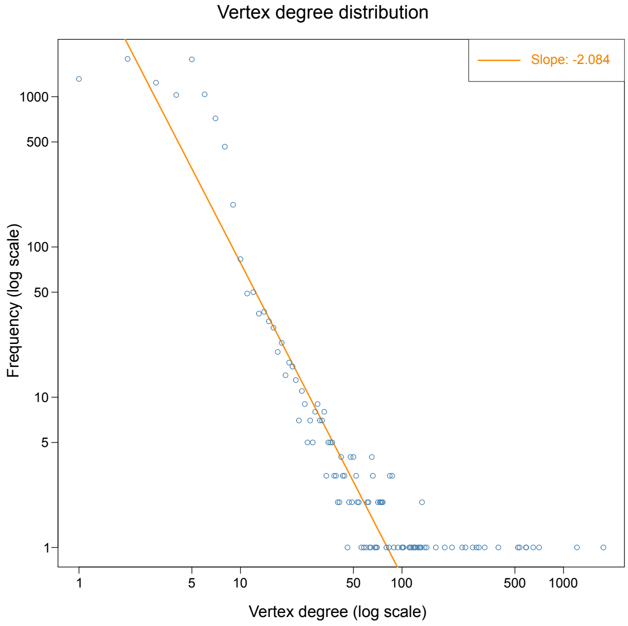

Figure D: Our KEGG graph degree distribution follows the scale-free network pattern $P(k) \sim k^{-\gamma}$, with $\gamma = 2.084 \in [2, 3]$. The heavy tail of this distribution confirms the existence of hubs, which are nodes with an extremely high degree and a major role in the biology.

Tenenbaum, D. (2016). *KEGGREST: Client-side REST access to KEGG*.
