# Supplementary material for: Null diffusion-based enrichment for metabolomics data
Source: PLoS One. 2017 Dec 6;12(12):e0189012. doi: 10.1371/journal.pone.0189012 (PMC5718512; doi:10.1371/journal.pone.0189012)
Supplement: S2 Appendix — Formulation of the heat diffusion scoring method. (PDF) [file pone.0189012.s005.pdf]

Appendix S2 - Heat diffusion process

The heat diffusion process is a model to quantify the propagation of flow in a network; this flow represents a biological perturbation when the experimental conditions change. However, note that this design is neither a functional model of biology nor a simulation of heat diffusion on biological molecules.

Using the explicit method for the finite difference formulation of the heat diffusion problem (Eq. 1), we can relate the temperatures between contiguous time instants in a meshed object containing n nodes. A graph can be naturally regarded as a meshed object, thus allowing the heat diffusion on our KEGG graph. The formulation (Bonals, 2005) is:

$$T^{k+1} = T^k + DTC \cdot [KI \cdot T^k + KC \cdot TC + G] \tag{1}$$

where

$$T^k = \begin{bmatrix} T_1^k \\ T_2^k \\ \vdots \\ T_n^k \end{bmatrix} {}^\circ C \tag{2a}$$

are the temperatures of the n nodes in the graph at the k-th instant, T_i^k. Also,

$$DTC = \begin{bmatrix} \frac{\Delta t}{C_1^k} & 0 & \dots & 0 \\ 0 & \frac{\Delta t}{C_2^k} & \dots & 0 \\ \vdots & \vdots & \ddots & \vdots \\ 0 & 0 & \dots & \frac{\Delta t}{C_n^k} \end{bmatrix} \frac{{}^\circ C}{W} \tag{2b}$$

is the diagonal matrix containing the quotient between the time step Δt, in seconds, and the heat capacity of node n at the k-th instant, C_n^k, in $\frac{J}{{}^\circ C}$. Following,

$$KI = \begin{bmatrix} -\sum_j K_{1j}^k & K_{12}^k & \dots & K_{1n}^k \\ K_{21}^k & -\sum_j K_{2j}^k & \dots & K_{2n}^k \\ \vdots & \vdots & \ddots & \vdots \\ K_{n1}^k & K_{n2}^k & \dots & -\sum_j K_{nj}^k \end{bmatrix} \frac{W}{{}^\circ C} \tag{2c}$$

contains the heat conductance between nodes v_i and v_j in the k-th instant, K_{ij}^k. The sums in the diagonal also account for the conductance to the boundary nodes if present. Next,

$$KC = \begin{bmatrix} K_{1,n+1}^k & K_{1,n+2}^k & \dots & K_{1,n+c}^k \\ K_{2,n+1}^k & K_{2,n+2}^k & \dots & K_{2,n+c}^k \\ \vdots & \vdots & \ddots & \vdots \\ K_{n,n+1}^k & K_{n,n+2}^k & \dots & K_{n,n+c}^k \end{bmatrix} \frac{W}{{}^\circ C} \tag{2d}$$

is the matrix containing the conductances from the node v_i to the l-th boundary node (which does not belong to the graph) in the k-th instant, $K_{i,n+l}^k$. As for these boundary nodes,

$$TC = \begin{bmatrix} T_{n+1}^k \\ T_{n+2}^k \\ \vdots \\ T_{n+c}^k \end{bmatrix} {}^\circ C \tag{2e}$$

is the vector that contains temperatures of the boundary node l in the k-th instant, T_{n+l}^k (note that there are c boundary nodes in total and that they are not in V). Finally,

$$G = \begin{bmatrix} G_1^k \\ G_2^k \\ \vdots \\ G_n^k \end{bmatrix} W \tag{2f}$$

contains the inner heat generation for node v_i in the k-th instant, G_i^k.

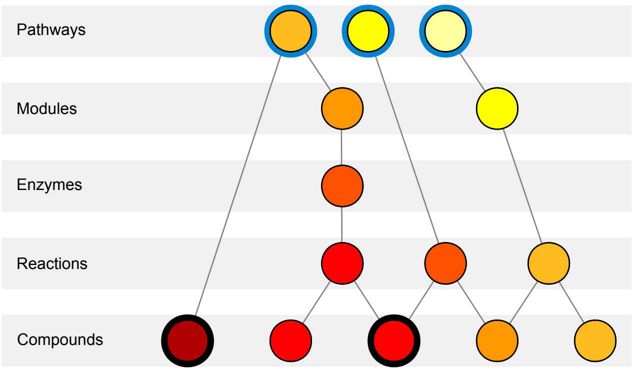

Figure A: Nodes arrangement for heat diffusion. In this tiny example, the levels represent the hierarchy from pathways (top) to compounds (bottom). The affected compounds are highlighted with a black ring. Affected compounds are forced to generate unitary flow. To reach a stationary state, these two flow units must evacuate through pathways, located at the top level. Every pathway is highlighted with a blue ring, representing its connection to a cool boundary node at 0°C. In the stationary state, depicted through heat colours proportional to the final temperature, the warmest pathways will hold greatest heat flow, suggesting a relevant role in the experiment.

The finite difference expression (Eq. 1) takes a substantially simpler form when applied to our node arrangement (Fig. A). First, imposing the stationary state $T^n = T^{n+1} = T$ and constant parameters for every time step:

$$T = -KI^{-1} \cdot [KC \cdot TC + G] \tag{3}$$

As shown in the proposed configuration (Fig. A), the boundary nodes are at 0°C, therefore the equation simplifies into Eq. (4).

$$T = -KI^{-1} \cdot G = R_{HD} \cdot G \tag{4}$$

where $R_{HD} = -KI^{-1}$ is the linear mapping of the heat diffusion process. This is the expression shown in the main body; an equivalent formulation can be found at HotNet (Vandin et al., 2011). The terms in the conductance matrix KI are given by the inverse of the weights in the curation process presented in Appendix S1, whereas conductances to boundary nodes are unitary. Besides allowing the calculation of temperatures, Eq. 4 also describes the diffusion process. For example, the null diffusion correlation matrix between biological entities in KEGG, described in Appendix S4, can give insights about the nature of the network.

Further analyses can be achieved through the conductance matrix of the graph (Bapat, 2004). This perspective is usually regarded as the electrical problem of finding the equivalent resistance between any couple of nodes. The resistance distance is a metric that takes into account all the possible paths from one vertex to the other, and not only its shortest path, thus effectively including the graph topology.

References

Bapat, R. (2004). Resistance matrix of a weighted graph. *Communications in Mathematical and in Computer Chemistry/MATCH*, 50:73–82.

Bonals, L. A. (2005). *Transferència de calor: apunts de classe.* Publicacions d'Abast.

Vandin, F., Upfal, E., and Raphael, B. J. (2011). Algorithms for detecting significantly mutated pathways in cancer. *Journal of Computational Biology*, 18(3):507–522.
