# Supplementary material for: Null diffusion-based enrichment for metabolomics data
Source: PLoS One. 2017 Dec 6;12(12):e0189012. doi: 10.1371/journal.pone.0189012 (PMC5718512; doi:10.1371/journal.pone.0189012)
Supplement: S3 Appendix — Formulation of the PageRank web ranking algorithm. (PDF) [file pone.0189012.s006.pdf]

Appendix S3 - PageRank

The PageRank algorithm (Page et al., 1999) scores every node in a graph using a web surfer model. The graph is typically directed because so are the hyperlinks between websites. Applying PageRank to an undirected graph is even more similar to the heat diffusion described in Appendix S3.

The web surfer model mimics the behaviour of real internet users. The surfer starts a random walk at a randomly chosen website, according to a prior probabilities vector p. In each step of the random walk, he or she decides whether to continue with the current random walk (probability d) or start a new one (probability $1-d$). If the random walk is resumed, the probability of choosing an edge is proportional to its weight. Finally, the PageRank scores are the stationary probability distribution over the graph nodes for this surfer.

Despite the different formulation of the PageRank problem, the final calculation of the PageRank scores is similar to the stationary state of our heat diffusion process. The arrangement of the nodes is identical (Fig. A), but the PageRank graph is directed upwards.

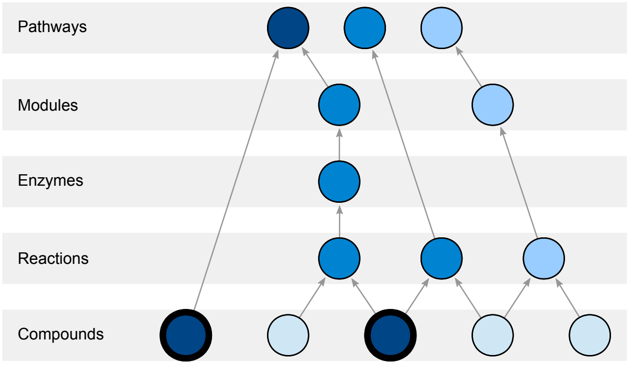

Figure A: Nodes arrangement for PageRank. Affected compounds are the start of random walks, uniformly distributed among them. PageRank scores, represented by the intensity of the blue colour, will attain larger values in the nodes that are frequently reached through the random walks in a stationary state. Random walks resumed in dead ends start in a uniformly chosen node from the graph.

The mathematical expression to obtain the scores (Equation 2) can be derived by imposing a stationary probability in the random walk process, whose website transitions are governed by the matrix M:

$$PR = d \cdot M \cdot PR + (1-d) \cdot p \tag{1}$$

obtaining

$$PR = R_{PR} \cdot p \tag{2}$$

p is the probability distribution for the source of new random walks and R_{PR} is the matrix:

$$R_{PR} = (1-d) \cdot (Id - d \cdot M)^{-1} \tag{3}$$

where Id is the identity matrix, d is the damping factor and M is a matrix obtained from the weighted adjacency $n \times n$ matrix A from the directed KEGG graph (edges pointing upwards):

$$M = f(t(A)) \tag{4}$$

In this expression, t is the matrix transpose operator and f is the function that normalises each column to sum 1, except when it contains n zeroes; in the latter case it returns a column with n elements equal to $\frac{1}{n}$. We apply the function f with that particularity to be coherent with the R package igraph (Csardi and Nepusz, 2006), which considers that terminal nodes resume the random walk uniformly distributed in all the vertices.

In Equation 2, the calculation of the PageRank scores uses the binary vector of affected compounds normalised by the amount of affected compounds (Fig. A):

$$p = \frac{G}{\sum G_i} \tag{5}$$

The similarity between the final expression for heat diffusion (see Appendix S2) and PageRank (Equation 2) is remarkable, given the common random walk background for these two methods. The differences between them include the forced upwards directionality of PageRank and the damping factor concept, which allows leaps in the diffusion. The rescaling of the p vector does not affect the null model, as the rescaling factor remains constant in the random trials.

All the PageRank scores in our approach have been computed using the standard $d = 0.85$ established in the original publication (Page et al., 1999), a range of damping factors has been swept, going from 0.1 (very frequent restarts) to 0.95 (almost no restarts), but results are consistent as a result of the application of our null model, described in Appendix S4.

References

Csardi, G. and Nepusz, T. (2006). The igraph software package for complex network research. *InterJournal, Complex Systems*, 1695(5):1–9.

Page, L., Brin, S., Motwani, R., and Winograd, T. (1999). The PageRank citation ranking: bringing order to the Web.
