# Supplementary material for: Null diffusion-based enrichment for metabolomics data
Source: PLoS One. 2017 Dec 6;12(12):e0189012. doi: 10.1371/journal.pone.0189012 (PMC5718512; doi:10.1371/journal.pone.0189012)
Supplement: S4 Appendix — Definition of the null models and visualisation of the pathway correlation matrix. (PDF) [file pone.0189012.s007.pdf]

Appendix S4 - Null models

We retrieve the formulation from Appendix S2 to compute the final temperatures in heat diffusion. The same procedure applies to the PageRank approach in Appendix S3, given the similarity between them, but as a proof of concept it will be developed for heat diffusion only.

$$T = R_{HD} \cdot G$$

By abuse of notation, R_{HD} will contain the columns of the original R_{HD} corresponding only to compounds, thus being a rectangular matrix from now on. Likewise, to have a well-defined matrix-vector product, G will only refer to compounds, as they are the only entities that can introduce heat. The vector G contains exactly n_{in} ones, corresponding to the affected compounds, and $n_{comp} - n_{in}$ zeroes, where n_{comp} is the amount of compounds in the graph.

When focusing on a node i, we want to assess whether its temperature T_i would be expected from a random selection of affected compounds or, on the contrary, the affected compounds are more related to node i than expected. To that end, we define the null distribution of temperatures:

$$T_{null} = R_{HD} \cdot X \tag{1}$$

where X is the random variable obtained by permuting G. If we define $p = \frac{n_{in}}{n_{comp}}$, then every X_i is a Bernoulli trial with success probability p. X_i and X_j, for $i \neq j$, are slightly anticorrelated due to the permutation approach.

Exact statistical moments of T_{null} can be computed:

$$\mathbb{E}(T_{null}) = R_{HD} \cdot \mathbb{E}(X) \tag{2}$$

being

$$\mathbb{E}(X) = p \cdot \begin{bmatrix} 1 \\ 1 \\ \vdots \\ 1 \end{bmatrix} \tag{3}$$

and the same for the covariance matrix

$$\Sigma(T_{null}) = R_{HD} \cdot \Sigma(X) \cdot R_{HD}^T \tag{4}$$

where

$$\Sigma(X) = p(1-p) \cdot \begin{bmatrix} 1 & \rho & \dots & \rho \\ \rho & 1 & \dots & \rho \\ \vdots & \vdots & \ddots & \vdots \\ \rho & \rho & \dots & 1 \end{bmatrix} \tag{5}$$

being $\rho = -\frac{1}{n_{comp}-1}$. In terms of the elements r_{ij} in matrix R_{HD}, we can write

$$\mu_i = \frac{n_{in}}{n_{comp}} \left[\sum_{j=1}^{n_{comp}} r_{ij} \right] \tag{6}$$

$$\sigma_i^2 = \frac{n_{in}(n_{comp} - n_{in})}{n_{comp}(n_{comp} - 1)} \left[\left(\sum_{j=1}^{n_{comp}} r_{ij}^2 \right) - \frac{1}{n_{comp}} \left(\sum_{j=1}^{n_{comp}} r_{ij} \right)^2 \right] \tag{7}$$

In fact, the correlation matrix of the null temperatures gives insights about the combination of the network structure and the null model. Focusing on pathways: if two pathways are strongly correlated, it suggests that both attain high or low temperatures with similar inputs. Thus, this couple of pathways are prone to overlap or to be nearby in the metabolism. Conversely, a strong anticorrelation suggests that warming up a pathway conditions the second pathway to become colder, suggesting that these pathways are dissimilar. (Fig. A) depicts

the correlations matrix for the pathways, where rows and columns have been reordered to illustrate clusters of KEGG pathways. Furthermore, the structure seems to be somehow related to the underlying biology: one of the clusters corresponds to the bulk of human metabolic pathways, whereas the genetic information processing pathways also appear highly correlated. Besides these examples, pathway types do not appear totally shuffled, but as small blocks of pathways sharing a biological role.

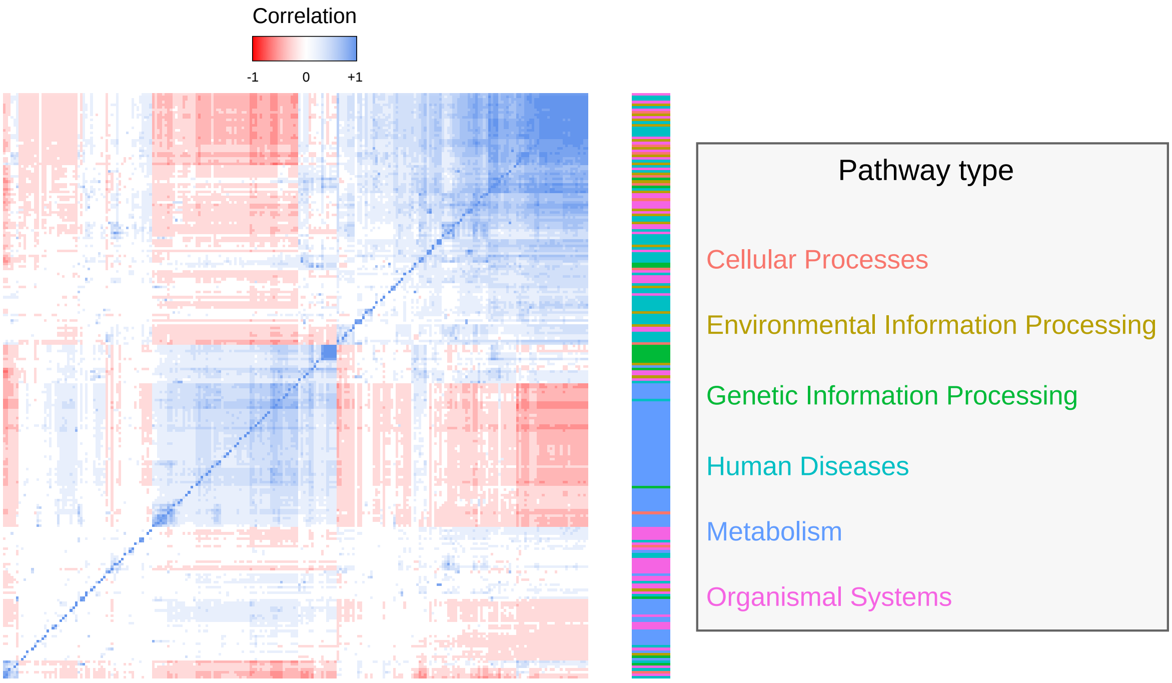

Figure A: Correlation matrix between pathways for the heat diffusion process in the KEGG graph (identifiers omitted for clarity). Blue correlations are closer to 1, while red tend to -1. For the calculation of these correlations, a count of 33 compounds was assumed to perform the null model, like in the experimental data. In addition, the biological role of the pathways annotated in KEGG BRITE (Kanehisa et al., 2008) has been drawn in the rightmost bar.

(Eqs. 6, 7) provide a first approximation **(1)** to evaluate how high a temperature is. If T_i is remarkably greater than $\mu_i = \mathbb{E}(T_{null})_i$ in terms of its standard deviation, $\sigma_i = \sqrt{\Sigma(T_{null})_{ii}}$, then node i should be reported. The normalised score is

$$z_i = \frac{T_i - \mu_i}{\sigma_i} \tag{8}$$

Another approach **(2)** to evaluate the relevance of node i is to perform the permutation analysis through Monte Carlo trials. In that case, the random vector X is drawn n_{perm} times and, for node i, the p-value is approximated as $p_i = \frac{r_i+1}{n_{perm}+1}$, where r_i is the number of trials where $T_{null_i} \geq T_i$, see (North et al., 2002) for further details on this estimator. An ensemble solution can be obtained by repeating the procedure n_{vote} times and evaluating each node by majority vote, specifically including it only if it is reported at least $\lfloor \frac{n_{vote}}{2} \rfloor + 1$ times, also allowing a fuzzy representation of the consensus solution. This ensemble approach reduces the variability in the reported solution and also provides confidence measures for each included node. The input can also be subsampled in this approach, although this option has not been explored yet.

References

Kanehisa, M., Araki, M., Goto, S., Hattori, M., Hirakawa, M., Itoh, M., Katayama, T., Kawashima, S., Okuda, S., Tokimatsu, T., and Yamanishi, Y. (2008). KEGG for linking genomes to life and the environment. *Nucleic Acids Research*, 36(Database-Issue):480–484.

North, B. V., Curtis, D., and Sham, P. C. (2002). A note on the calculation of empirical P values from Monte Carlo procedures. *American Journal of Human Genetics*, 71(2):439.
