# Supplementary material for: Null diffusion-based enrichment for metabolomics data
Source: PLoS One. 2017 Dec 6;12(12):e0189012. doi: 10.1371/journal.pone.0189012 (PMC5718512; doi:10.1371/journal.pone.0189012)
Supplement: S5 Appendix — Solution stratification, CC evolution, computational cost of Monte Carlo permutations and damping factor influence. (PDF) [file pone.0189012.s008.pdf]

Appendix S5 - Details on reported solutions

The solutions reported in the main body encompass two different scoring functions (heat diffusion and PageRank) and two statistical approaches (z-scores and simulation). Monte Carlo permutations involve consensus solutions among the simulated runs to reduce the variability and enhance robustness and consistency between runs.

Solution stratification

(Fig. A) depicts the stratification of all the reported graphs. Solutions tend to keep the same proportions as the original graph, allowing the discovery of relevant nodes in all the categories. This is not only a sign of agreement across the different solutions, but also a necessary behaviour to discover putative nodes in all the categories. The proportion of reported compounds seems lower than the one in the KEGG graph, probably due to the application of the null model, which tends to favour the metabolites in the input and penalise the rest.

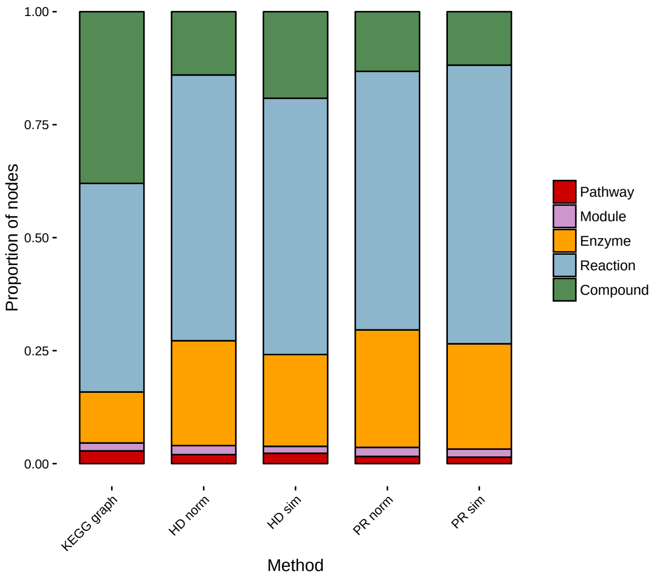

Figure A: Subgraph stratification by method. Notice the tendency to keep the same distribution as the whole KEGG graph. Compared to the KEGG graph, the proportion of compounds decreases in all the solutions due to the inclination to recover the ones in the input and exclude the rest.

Connected component evolution

The choice of the number of desired nodes k affects the number and size of the reported connected components (CC). In general terms, the number of reported CCs seems to lower as the number of reported nodes grows (Fig. B), as different CCs that contain seed nodes tend to merge. The number of nodes in the largest CC grows monotonically as k increases and it captures the majority of the nodes in the subgraphs (Fig. C).

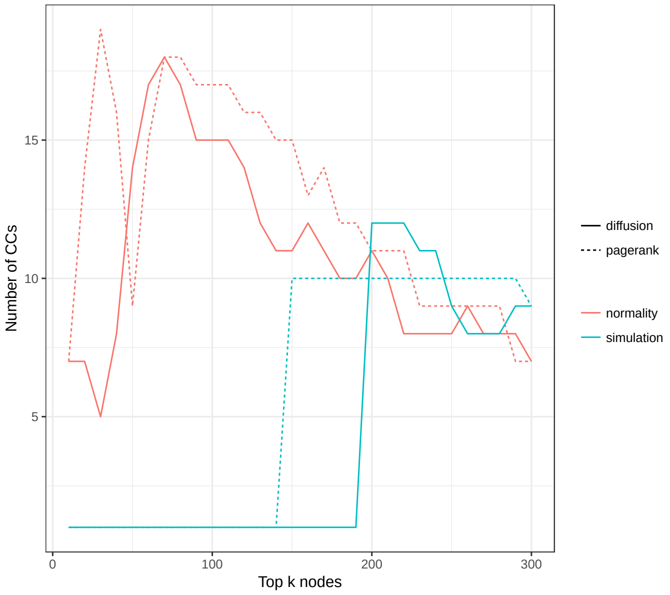

Figure B: Number of reported CCs for varying k. The discrete nature of the majority vote approach in simulation trials leads to ties, which can be spotted as horizontal lines in the figure - more than a hundred nodes are tied with the best rank in both cases. In general, the number of CCs decreases as the reported subgraphs grow.

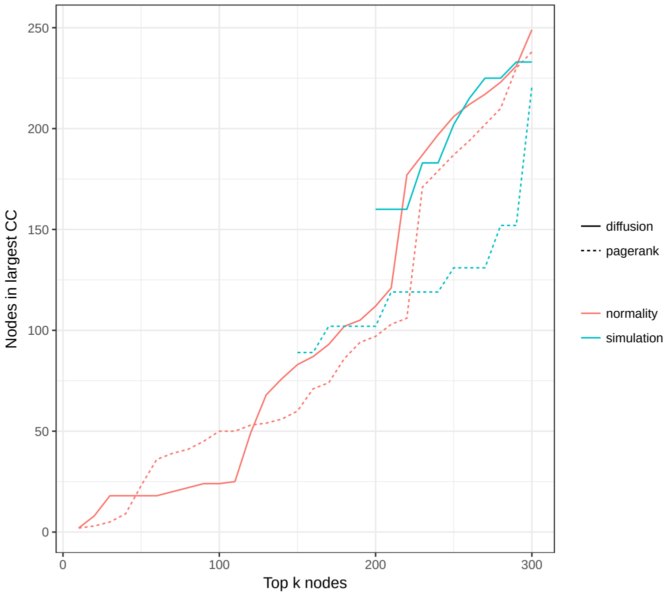

Figure C: Number of nodes in the largest CC reported. If more nodes are reported, the largest CC grows accordingly. For $k = 300$ around 225-250 nodes are in the largest CC in every approach, meaning that approximately 75-83% nodes lie in it. As more than a hundred nodes are tied with maximum rank in the simulated version, lines start at these respective points.

Computational cost

In sight of further applications of these methodologies, we have performed a benchmark of several implementations of the Monte Carlo approach. For heat diffusion, the temperature calculation (Appendix S2) is achieved through

$$T = -KI^{-1} \cdot G = R_{HD} \cdot G \tag{1}$$

where G has n_{in} ones and $n_{comp} - n_{in}$ zeroes.

We define two strategies to permute the input G: **(a)** draw n_{in} elements without replacement from the set $[1, n_{comp}]$, and **(b)** shuffle the whole vector G. Furthermore, two possible calculations for T are: **(1)** explicitly compute R_{HD} and sum the columns indexed by the n_{in} ones, or **(2)** solve the linear system $T = -KI^{-1} \cdot G$

These strategies have been essayed with growing graph order, from 1,000 to 10,000 nodes. Graphs are randomly generated using the default Barabási-Albert model in igraph (Csardi and Nepusz, 2006). Then, 10% of the nodes are randomly selected to be pathways, so the heat flow can be dispelled.

We also consider two scenarios, depending on if **(I)** the input list has a fixed size of 30 compounds, or **(II)** it scales as 10% of the graph nodes. For each combination of parameters, a benchmark of 30 permutations is run 10 times and the trends are depicted in (Fig. D). The differences between sampling strategies seem irrelevant compared to the solving method. Solving the linear system seems a good option for small inputs that do not scale with the graph order, but computing the inverse seems to scale better as the vector G becomes less sparse. However, the latter requires a vast amount of memory to store the matrix, so the best implementation will depend on the graph order and memory availability. All the benchmarks have been executed in a desktop workstation (Intel i5 650 at 3.2GHz, 16Gb RAM memory).

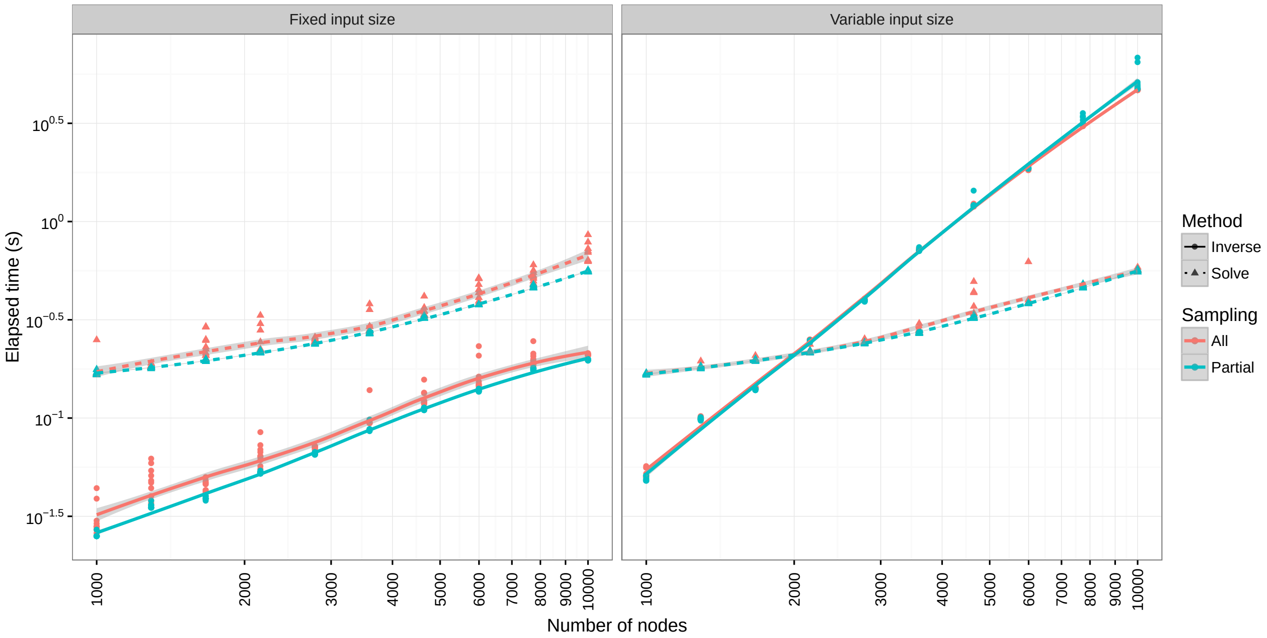

Figure D: Computational cost of several strategies for computing 30 permutations, that is, 30 null temperatures for all the nodes. These simulations have been performed 10 times with each combination of parameters. In the left figure, the input size is kept constant and equal to 30 compounds, whereas in the right it scales with the graph nodes (10%). Two methods to compute the temperatures are compared: direct resolution (solve) and explicit computation of the R_{HD} matrix (inverse). Likewise, two sampling strategies are explored: permute the whole G vector (all) or just draw the n_{in} compounds (partial).

Damping factor influence

The damping factor in the PageRank setup (see Appendix S3) is a model parameter that could affect the results if set differently. Although the standard value $d = 0.85$ was used, we analysed the parameter sensibility by sweeping several values of d, computing the z-scores and reporting the top 250 nodes (Figs. E, F). The normalised scores seem stable in a wide range of choices of d, therefore its choice does not seem a critical issue.

References

Csardi, G. and Nepusz, T. (2006). The igraph software package for complex network research. *InterJournal, Complex Systems*, 1695(5):1–9.

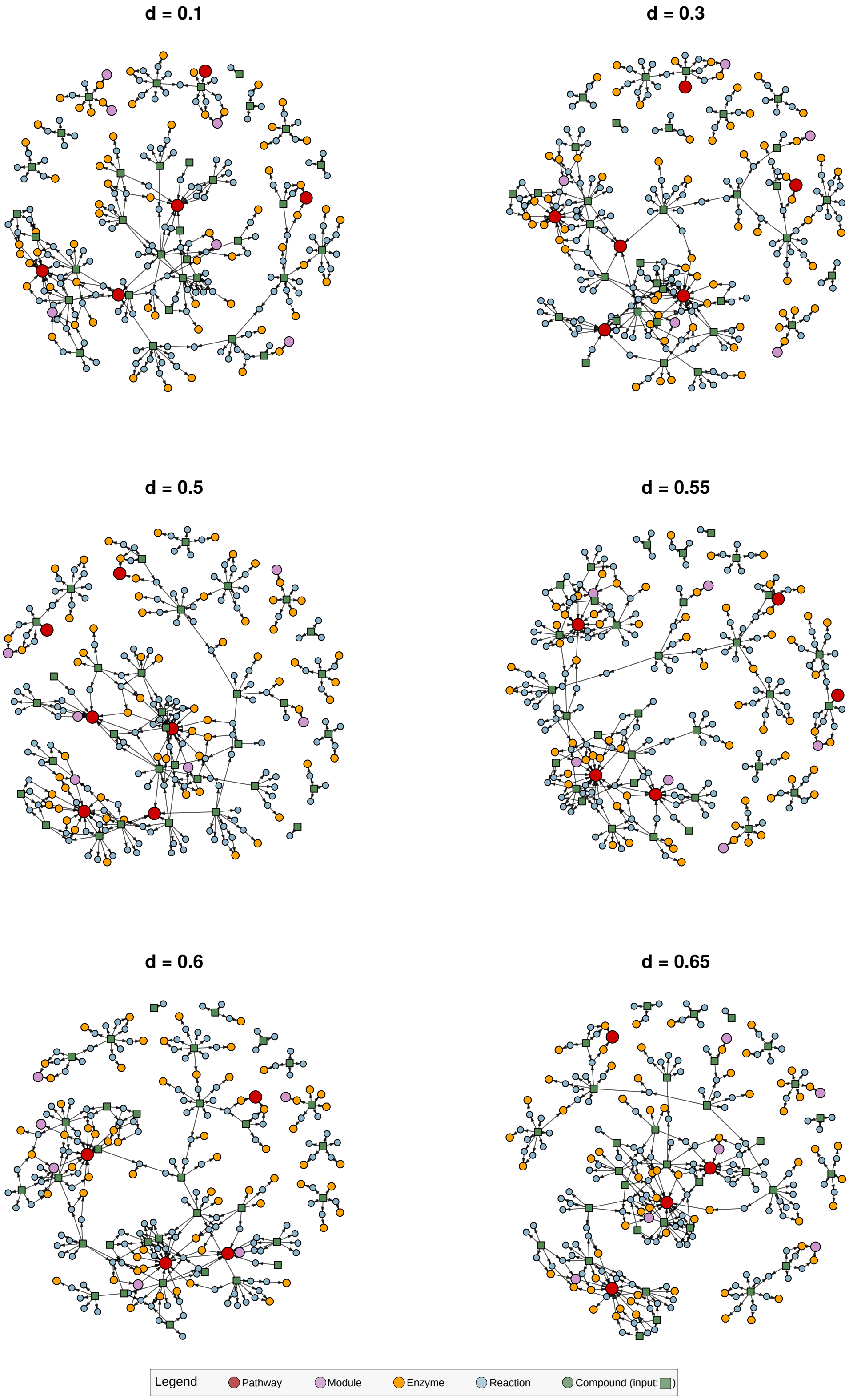

Figure E: Damping factor impact. The normalised z-scores show consistent solutions for a range of damping factors.

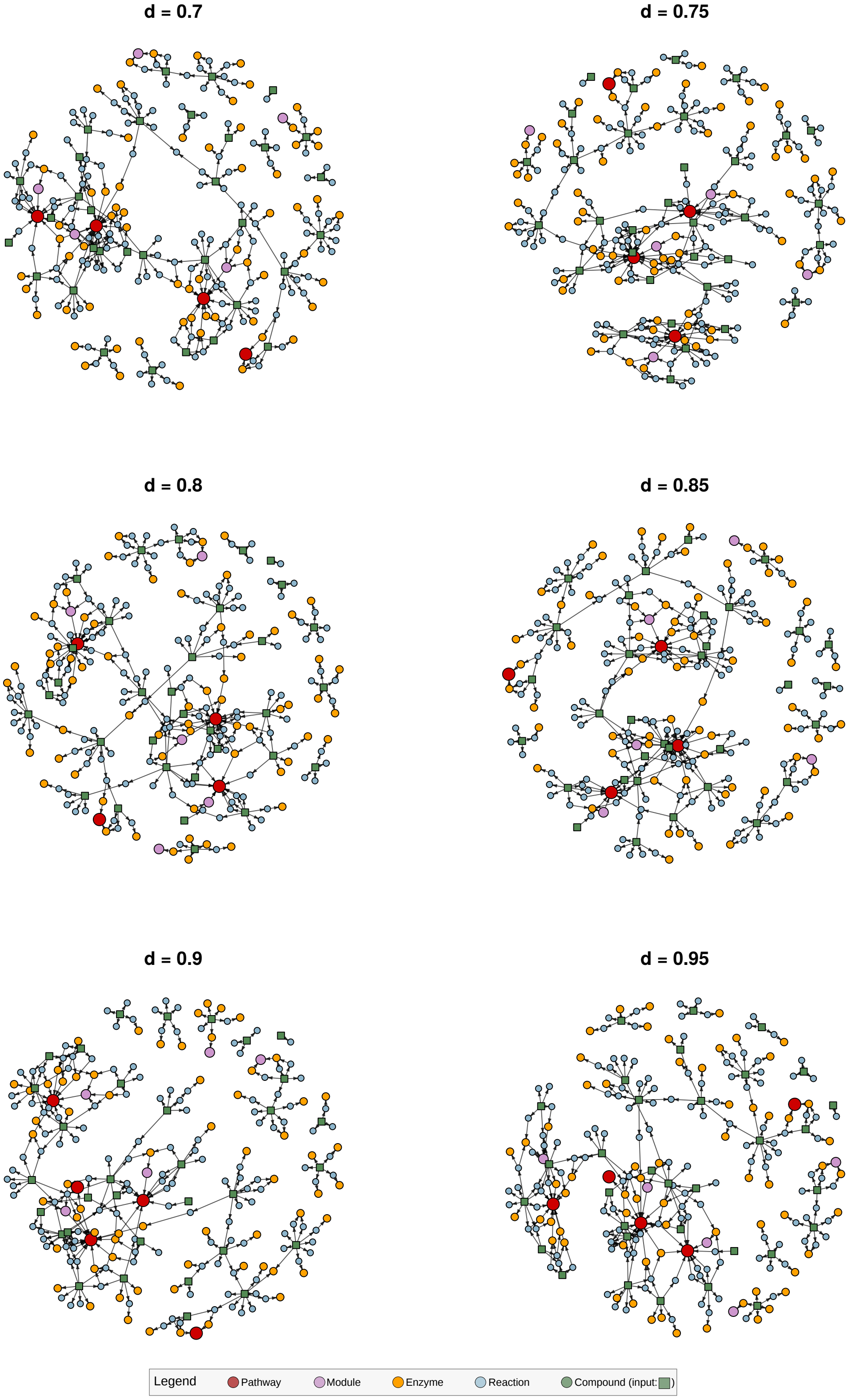

Figure F: Damping factor impact (continued). The normalised z-scores show consistent solutions for a range of damping factors.
